# Genome structure and diversity among *Cynanchum wilfordii* accessions

**DOI:** 10.1186/s12870-021-03390-y

**Published:** 2022-01-03

**Authors:** Sae Hyun Lee, Jiseok Kim, Hyun-Seung Park, HyunJin Koo, Nomar Espinosa Waminal, Remnyl Joyce Pellerin, Hyeonah Shim, Hyun-Oh Lee, Eunbi Kim, Jee Young Park, Hong Seob Yu, Hyun Hee Kim, Jeonghoon Lee, Tae-Jin Yang

**Affiliations:** 1 Department of Agriculture, Forestry and Bioresources, Plant Genomics & Breeding Institute, College of Agriculture & Life Sciences, Seoul National University, 1 Gwanak-ro, Gwanak-gu, Seoul, 08826 Republic of Korea; 2 Department of Life Sciences, Chromosome Research Institute, Sahmyook University, Seoul, 01795 Republic of Korea; 3 Phyzen Genomics Institute, Seongnam, 13558 Republic of Korea; 4 National Institute of Horticultural and Herbal Science, RDA, Eumseong, 27709 Republic of Korea

**Keywords:** *Cynanchum wilfordii*, *C. Auruculatum*, Diversity, Karyotype, Genome size

## Abstract

**Background:**

*Cynanchum wilfordii* (Cw) and *Cynanchum auriculatum* (Ca) have long been used in traditional medicine and as functional food in Korea and China, respectively. They have diverse medicinal functions, and many studies have been conducted, including pharmaceutical efficiency and metabolites. Especially, Cw is regarded as the most famous medicinal herb in Korea due to its menopausal symptoms relieving effect. Despite the high demand for Cw in the market, both species are cultivated using wild resources with rare genomic information.

**Results:**

We collected 160 Cw germplasm from local areas of Korea and analyzed their morphological diversity. Five Cw and one Ca of them, which were morphologically diverse, were sequenced, and nuclear ribosomal DNA (nrDNA) and complete plastid genome (plastome) sequences were assembled and annotated. We investigated the genomic characteristics of Cw as well as the genetic diversity of plastomes and nrDNA of Cw and Ca. The Cw haploid nuclear genome was approximately 178 Mbp. Karyotyping revealed the juxtaposition of 45S and 5S nrDNA on one of 11 chromosomes. Plastome sequences revealed 1226 interspecies polymorphisms and 11 Cw intraspecies polymorphisms. The 160 Cw accessions were grouped into 21 haplotypes based on seven plastome markers and into 108 haplotypes based on seven nuclear markers. Nuclear genotypes did not coincide with plastome haplotypes that reflect the frequent natural outcrossing events.

**Conclusions:**

Cw germplasm had a huge morphological diversity, and their wide range of genetic diversity was revealed through the investigation with 14 molecular markers. The morphological and genomic diversity, chromosome structure, and genome size provide fundamental genomic information for breeding of undomesticated Cw plants.

**Supplementary Information:**

The online version contains supplementary material available at 10.1186/s12870-021-03390-y.

## Background

*Cynanchum wilfordii* (Cw) and *Cynanchum auriculatum* (Ca) are perennial plants with climbing vines and tuberous roots that are used in traditional medicine in Korea and China, respectively. The dried roots of Cw and Ca are called “Baeksuo” and “Bai shou wu” in Korea and China, respectively, and are known to be high in nutrients, to have detoxifying effects, and to promote digestion. Scientific investigations have revealed various medicinal effects of Cw and Ca, particularly anti-inflammatory, antineoplastic, and anti-oxidative effects, reduction of depression, and relief of menopausal symptoms [28].

Cw and Ca are closely related species with similar medicinal effects. Cw is one of the best-known herbal plants used as a functional food in South Korea, and its cultivation area increased sharply from 2013 to 2016 (http://kostat.go.kr). Meanwhile, Ca was introduced into Korea approximately 30 years ago from China and is now also cultivated by some farmers in South Korea. These two species are similar in morphology and metabolite compositions. Distinguishing between Cw and Ca based on the morphological differences of their roots is especially difficult when the root skin has been peeled off for commercial sale. In 2015, concerns about economically motivated adulteration (EMA) arose in Korea over the substitution of Ca, which is not recognized as a medicinal plant, for Cw in functional foods [9].

Molecular markers used to investigate EMA issues are typically developed from plastome sequences and the sequences of nuclear ribosomal (nr) RNA internally transcribed spacers 1 and 2 (ITS1 and ITS2). Most polymorphic sites in plastomes and nrDNA genomes are found at the interspecies level, making them useful barcoding targets for analyses of genetic diversity, evolution, and phylogenetic relationships between species [14]. However, intraspecies diversity also has been identified in several medicinal plants and provides a useful classification system in, for example, *Panax ginseng* [26, 27], *Peucedanum japonicum* [19], *Lonicera japonica* [12], and *Rehmannia glutinosa* [11].

Cw is an indigenous resource plant in Korea and has been cultivated by farmers for several decades. The cultivated plants are derived from seeds collected from wild plants that are not yet domesticated. Breeding efforts were initiated only recently, and there is no officially registered cultivar in Korea. Therefore, farmers currently cultivate an admixture of wild local accessions. In this context, some previously reported species-specific markers produce confusing results due to intraspecies variation or mitochondrial plastid genome (MTPT) interference in plastid marker application. In addition, the occurrence of EMA has been invoked on the basis of only one or two DNA markers, which could result in false-positives for genuine Cw products resulting from the application of DNA markers derived from the MTPT in conjunction with the genetic diversity of Cw accessions [29].

In this study, we investigated the diverse morphological characteristics along with the genome size and chromosome structure of Cw and Ca, with the goal of exploring the genetic diversity of these two species and providing basic genome information that can be used in breeding programs. We obtained plastomes and sequences of the complete 45S nrDNA and 5S nrDNA units from five Cw accessions and two Ca accessions and identified interspecies plastome diversity between Cw and Ca and intraspecies plastome diversity among Cw accessions collected in Korea. We also developed DNA markers and examined plastome genotypes among wild Cw accessions. Our data will help support the breeding, evaluation of genetic diversity, and classification of Cw accessions.

## Results

### Cw showed morphological diversity and compact genome structure

**Fig. 1:**
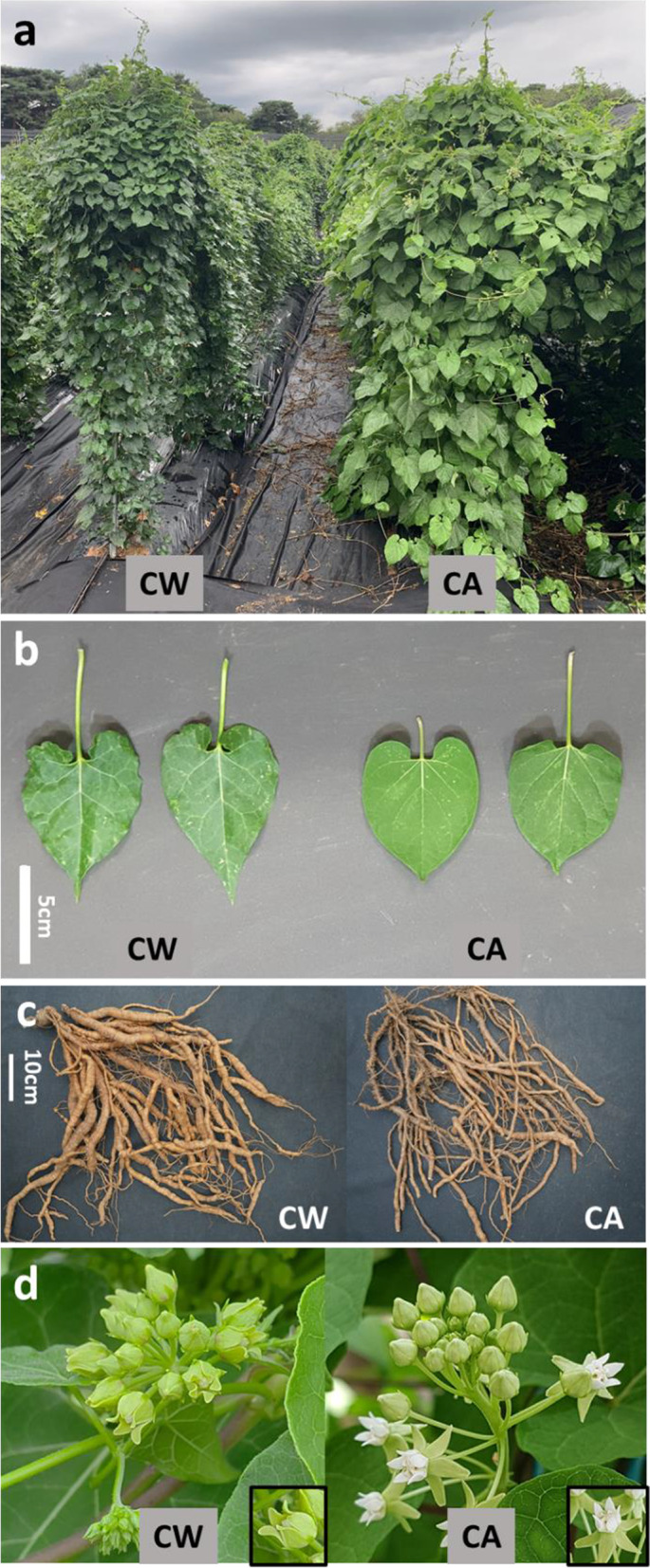
Morphology of Cw and Ca plants. **a** Vines grown in the field for 2 years. **b** Typical leaf shapes. (Waminal et al.) Root shapes. (Waminal et al.) Flower shapes. Cw flowers are hidden by sepals and do not open fully even at maturity, whereas Ca flowers are exposed by the wide opening of sepals, which are completely rolled back

**Fig. 2:**
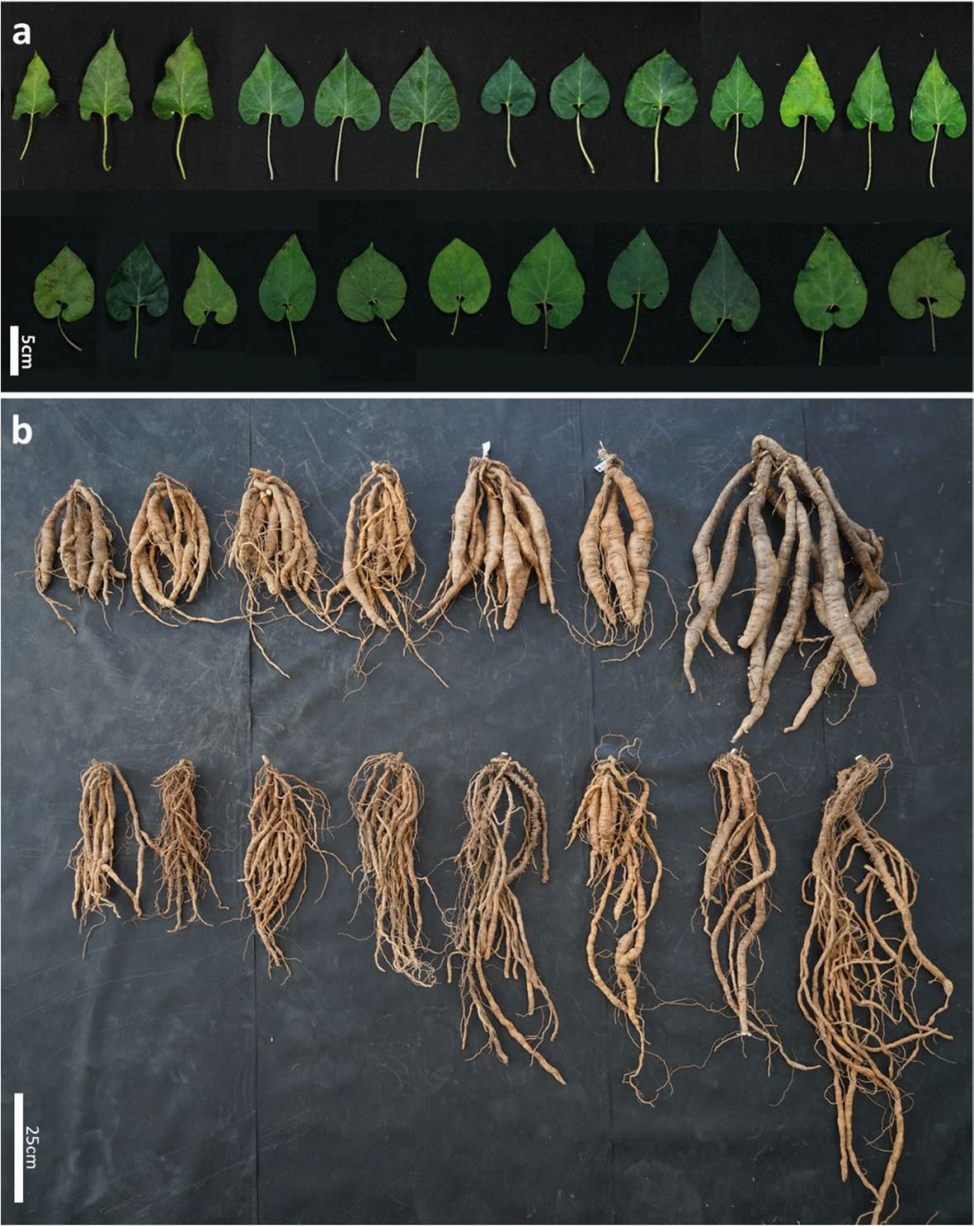
Morphological diversity of leaves and roots among Cw germplasms. **a** Diverse shape, colors and sizes of Cw leaves cultivated during 1 year in the field. **b** Harvested Cw roots have grown in the field for 1 year

The two *Cynanchum* species can be clearly identified on the basis of flower shape because Ca has a rolled-back calyx, whereas Cw has a calyx that covers the petals (Fig. 1). However, the leaves and roots are similarly shaped in both species. We cultivated 160 Cw accessions and measured their morphological diversity. Most Cw accessions had standard heart-shaped leaves, but some had unique leaves with sharp points, and it is completely deviated from the common shape of Cw leaves (Figs. 1 and 2b). Root length, thickness, and weight varied from 8.5 to 93.5 cm, 4.5 to 40.5 mm, and 15 to 795 g, respectively (Figs. 2, S1, S2). Overall, the Cw population was extremely diverse in morphological traits.

**Fig. 3:**
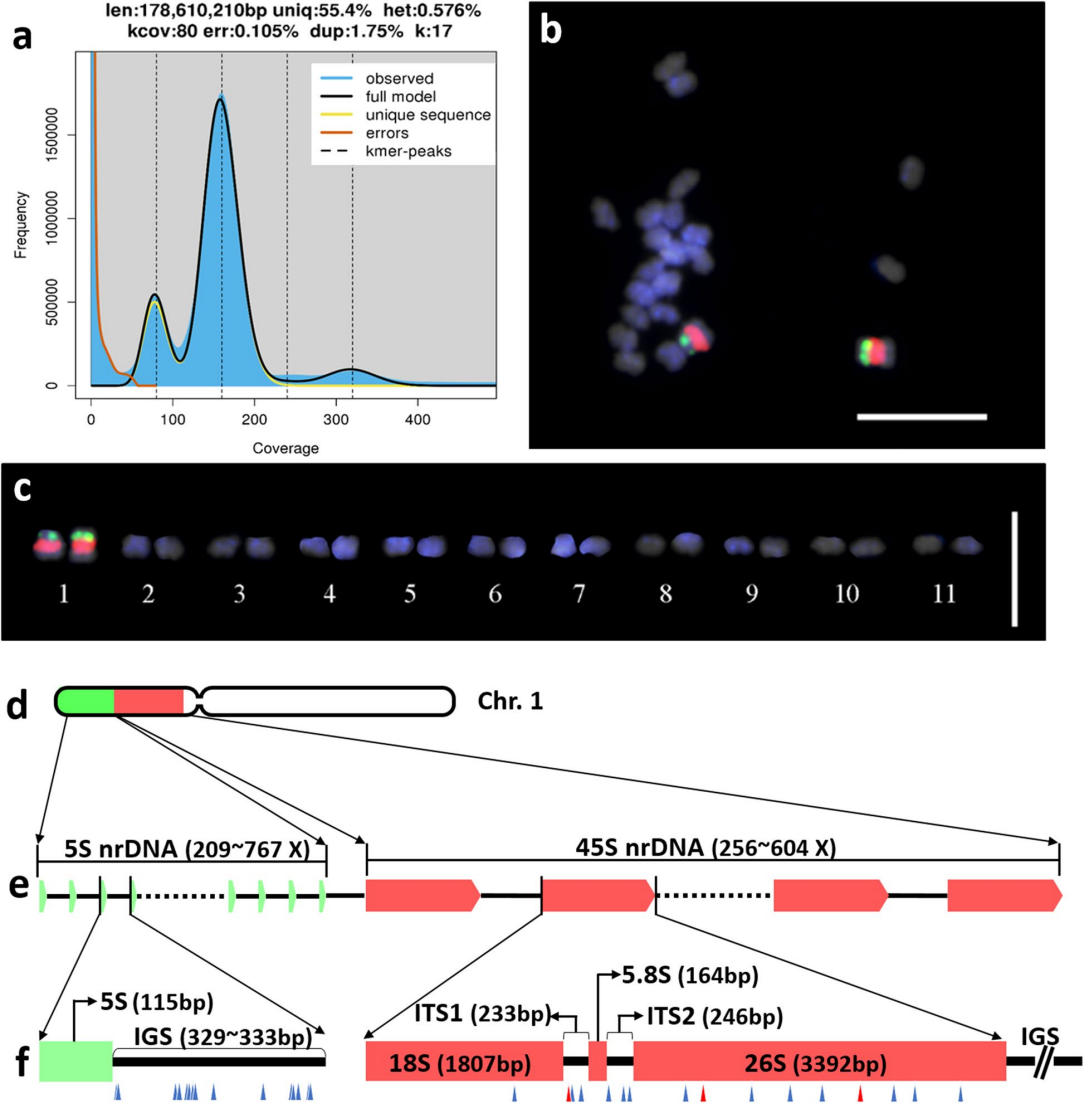
Genome size and chromosome structure of Cw. **a** Distribution plot of *k-*mer analysis using 17-mers. **b** Cw chromosome spread stained with DAPI and 5S nrDNA and 45S nrDNA probes. The 5S and 45S nrDNA are represented as green and red signals, respectively. Bar, 10 μm. (Waminal et al.) Eleven chromosome pairs displayed by size and shape. Chromosomes are arbitrarily lined up by size; bar, 10 μm. (Waminal et al.) Relative positions of 45S and 5S nrDNA blocks on chromosome 1. **e** Schematic diagram of the 45S and 5S transcription units clustered separately. Green boxes represent 5S nrDNA, and red boxes represent 45S nrDNA arrays. The number of copies presumed to be in one cell (Table S2) is listed above. **f** Sequence information for 5S and 45S nrDNA units. Green rectangles represent 5S; blue triangles indicate interspecific SNPs and InDels (Table S5). Red rectangles represent 18S, 5.8S, and 26S nrRNA, blue triangles indicate interspecific variations between Cw and Ca, and red triangles indicate three interspecific variations that coincide with intraspecific variations found in Cw (Table S5)

We estimated the genome size of Cw based on *k*-mer analysis using 160 Gb of Whole-genome shotgun sequences (WGS) data. Double peaks were observed in the *k*-mer graph which showed a high heterozygosity rate. We estimated the Cw genome size to be approximately 178 Mbp, with 0.578% of heterozygosity. Cytogenetic analysis based on Fluorescence in situ hybridization (FISH) analysis using ribosomal DNA revealed 11 pairs of tiny chromosomes displaying typical diploid characteristics; one 5S tandem array block and one 45S nrDNA tandem array block were located in parallel on the same chromosome (Fig. 3b, c, d). To our knowledge, this is the first report of the chromosome shape and genome size for this plant.

### Sequence assembly and annotation of plastomes and nrDNA

Among the 160 accessions, we selected five Cw individuals showing diverse morphology for further sequencing analysis. WGSs were generated from five Cw individuals and one Ca individual. Complete plastomes were assembled in our previous research, and nrDNA sequences were assembled using the dnaLCW method [14, 15, 29] (Table S2). Cw plastomes ranged from 160,829 to 161,241 bp in length, showing the typical quadripartite structure with a large single copy of 91,606 to 91,642 bp, a short single copy of 19,871 to 19,907 bp, and a pair of inverted repeats (IRB and IRA) of 24,658 bp (Table S2). The Cw plastomes consisted of 137 genes, of which 100 were protein-coding genes and the others were transfer RNA or ribosomal RNA genes. We calculated the number of plastome copies in a cell using the estimated 356-Mbp diploid genome size as 432–799 (mean 597).

We simultaneously assembled 45S and 5S nrDNA sequences using the same WGS data. The 45S nrDNA and 5S nrDNA sequences of all Cw and Ca individuals were 5820 and 115 bp, respectively. The 45S nrDNA transcription unit comprised five parts: 18S (1807 bp), 5.8S (164 bp), and 26S (3392 bp) ribosomal RNA genes and two internal transcribed spaces, ITS1 (233 bp) and ITS2 (246 bp) (Fig. 3f, Table S2). The 5S nrDNA transcription unit comprised a 115-bp 5S nrRNA and a 329- to 333-bp intergenic spacer (IGS) (Fig. 3f). There were 256–604 (mean 416) and 209–767 (mean 428) 45S nrDNA and 5S nrDNA copies, respectively, in the 178-Mbp haploid genome (Fig. 3e, Table S1). The diverse complete sequences of plastome and nrDNAs provide resources for genome diversity for both species, and the estimated copy number provides basic genome information.

### Cw and Ca exhibit interspecies genome diversity

We identified 253 InDels and 973 SNPs between Cw and Ca based on intensive comparison of seven plastomes (5 Cw and 2 Ca) (Table S3). Almost half of the InDels were derived from copy number variation (CNV) of tandem repeats (TR). We identified 115 TRs across the Cw and Ca plastomes, among which 88 showed CNV, and 26, 7, and 55 were found in coding sequences (CDSs), introns, and intergenic regions, respectively. Of the TRs, 56 showed CNV in both species, while 12 and 11 TRs were unique to Cw and Ca, respectively. Most TRs were located in the intergenic region. The species-specific repeat units found in CDSs were TR45, TR84, and TR105 in Cw and TR106 in Ca. These TRs were found in the genes *accD* and *ycf1*. The longest TR was 63 bp, located in the intergenic region between *trnE-UUU* and *trnT-GGU*. The TR with the highest copy number (21 copies) was located in the *accD* gene region (Table S4). We also identified 16 SNPs between Cw and Ca 45S nrDNA. Three of these SNPs, one in ITS1 and two in the 26S region, coincided with SNPs identified among the 45S nrDNA of the five Cw accessions (Fig. 3f, Table S5). The 5S nrDNA did not show any divergence, but the IGS regions contained 13 SNPs and two InDels between the two species (Fig. 3f, Table S6). The genetic diversity identified in plastomes and nrDNA provide a genetic tool for molecular identification of these two species.

### Cw accessions show considerable intraspecies genome diversity

**Fig. 4:**
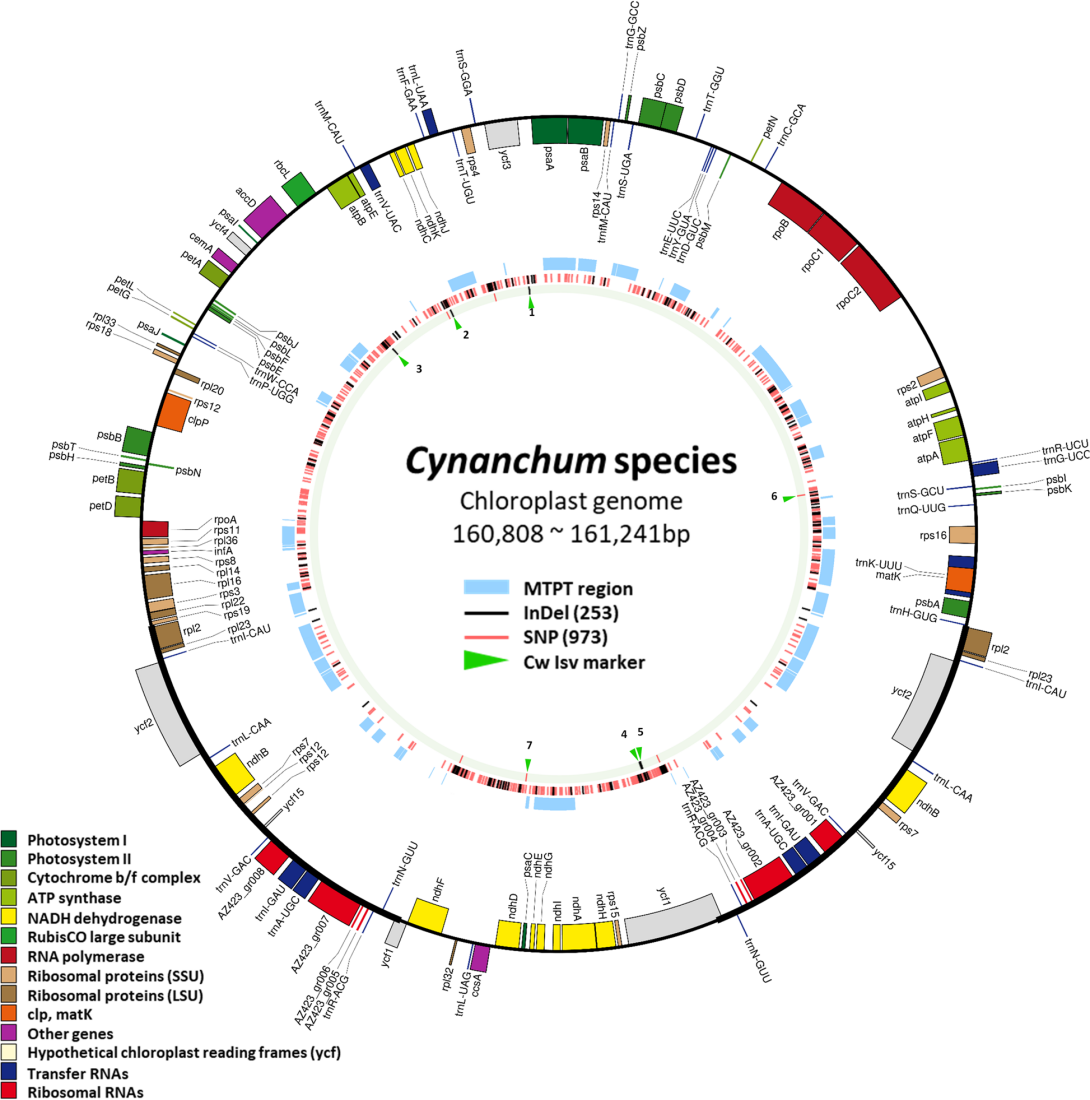
Complete plastome structure of *Cynanchum* species and schematic diagrams of inter- and intraspecies variable regions showing the position of DNA markers. On the outermost circle, colored boxes show conserved chloroplast genes classified according to product function. Genes shown on the outside of the map are transcribed clockwise, while those on the inside are transcribed counter-clockwise. In the inner circles, from inside to outside, the circles indicate the location of Cw intraspecies variation, interspecies variation between Cw and Ca, and the MTPT, respectively. Black and pink signals represent the positions of InDels and SNPs, respectively. Green arrowheads indicate the location of intraspecies variation markers; 1–7 represent isv1, isv2, isv3, isv4, isv5 (Table S7), isv_cp_kasp1, and isv_cp_kasp2 (Table S8), respectively

**Fig. 5:**
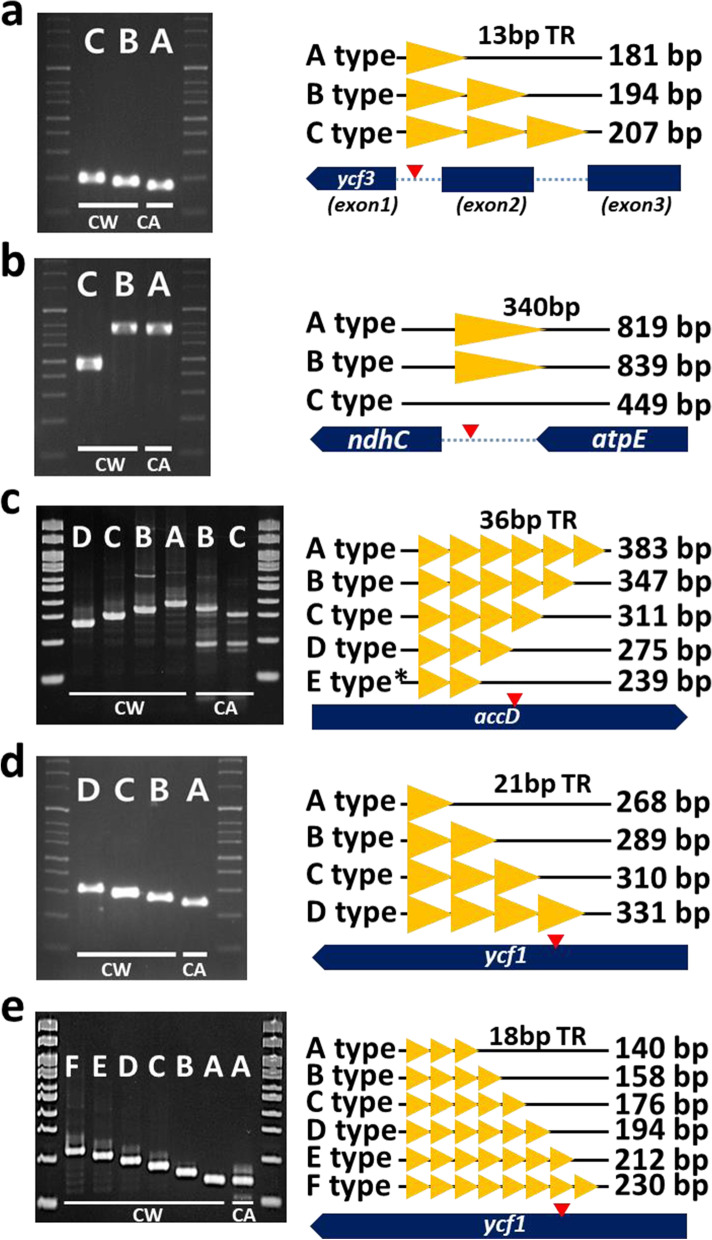
Amplification of diverse alleles and schematic representation of five DNA markers. **a–e** Five intraspecific markers designed using regions of Cw intraspecies diversity applied to the Cw and Ca populations (**a**: isv1, **b**: isv2, **c**: isv3, **d**: isv4, **e**: isv5). Primer information is shown in Table S7. Genotyping of each band is based on size differences, labeled alphabetically. Stained electrophoretic gels showing representative genotypes for each marker are displayed at left. Schematic diagrams of marker positions in the genome (red arrows), repeat motifs (yellow arrowheads), and PCR product sizes for each genotype are given at right. The types detected in the population, although not observed in the sequence data, are marked with an asterisk

We also identified five InDels and six SNPs showing intraspecies variation among the five Cw plastomes (Figs. 4 and 5, Table S7). Three of the five InDels were derived from genic CDSs (isv3 in CDS of *accD*, and isv4 and isv5 in CDS of *ycf1*), and the other two were found in the intron of *ycf3* (isv1) and the intergenic region between *ndhC* and *atpE* (isv2) (Fig. 5)*.* Four of the five were derived from TR CNVs, and isv2 was derived from a 340-bp deletion in four of the Cw plastomes (Fig. 5). The smallest repeat unit size was 13 bp and the largest was 36 bp (Table S4). Four of the six SNPs were located in the intergenic region (Fig. S3b, c, d, f), and the others were the intron of *trnG-*UCC and the CDS of *ndhD*, respectively (Fig. S3a, e). The point mutation in *ndhD* caused a synonymous mutation.

To examine plastome diversity in Cw population, we designed five InDel markers and two Kompetitive allele-specific PCR (KASP) markers suitable for determining polymorphisms (Tables S7, S8). The 160 Cw germplasm accessions collected from various regions of Korea showed diverse genotypes, with markers identifying two to six different alleles. However, Ca populations introduced from China were highly homogeneous, with only one marker revealing two different alleles (Fig. 5). We genotyped the 160 Cw accessions for each of the seven markers. The accessions could be divided into 21 plastome haplotypes, with two large groups classified by markers isv2 and isv_cp_kasp2 and further divided by isv1, isv3, isv4, isv5, and isv_cp_kasp1. Group Cp-I was divided into three subgroups composed of 14 unique genotypes, and group Cp-II comprised two subgroups containing seven unique genotypes (Fig. 7, Table S9).

We also developed seven SNP markers from nuclear genic regions based on sequence diversity between homologous genes found in the five WGSs. These seven markers classified the 160 Cw individuals into six major groups, with group Nc-II further classified into two subgroups. Between 20.62 and 43.75% of the individuals had heterozygous genotypes for each marker, and we identified 108 unique genotypes among the 160 accessions (Fig. 7). The two large plastome haplotypes, Cp-I-1 and Cp-II-1, were scattered sporadically on the nuclear-genome-based tree (Fig. 7). The seven nuclear-based markers showed an average of 0.343 and 0.349 observed heterozygosity (*H*_o_) and expected heterozygosity (*H*_e_) among the Cw accessions, respectively (Table S10). The plastome and nuclear markers will provide for genetic diversity among the Cw population.

### The CW population exhibits hypervariable (HV) plastome genes

One InDel marker, isv5, was found in the HV *ycf1* gene and showed six different genotypes among the Cw accessions. We identified eight TRs in *ycf1*, which generated polymorphism between Cw and Ca and also diverse genotypes based on CNVs among Cw accessions (Figs. 5 and 7). We also identified HV regions in the *accD* gene (Fig. S4). No variations were observed in the core functional regions such as the binding sites of acetyl-CoA or carboxybiotin or the CoA-carboxylation catalytic site (Fig. S5). We identified a long stretch of four TR types sharing the first 13 bp of the repeat motif. TR45 and TR46 shared the same position and had the same length but different motifs (Fig. S4, Table S4). The CNVs of the TRs were richer than our expectation based on comparison of the five Cw plastomes. PCR marker-based genotyping of the TRs uncovered novel alleles with more abundant copy numbers among the 160 Cw accessions (Figs. 5 and 7, S4)*.* The PCR markers derived from the HV regions will be useful for the sub-grouping of wild Cw collections.

## Discussion

### HV repeats in CDS of *accD* and *ycf1* genes

**Fig. 6:**
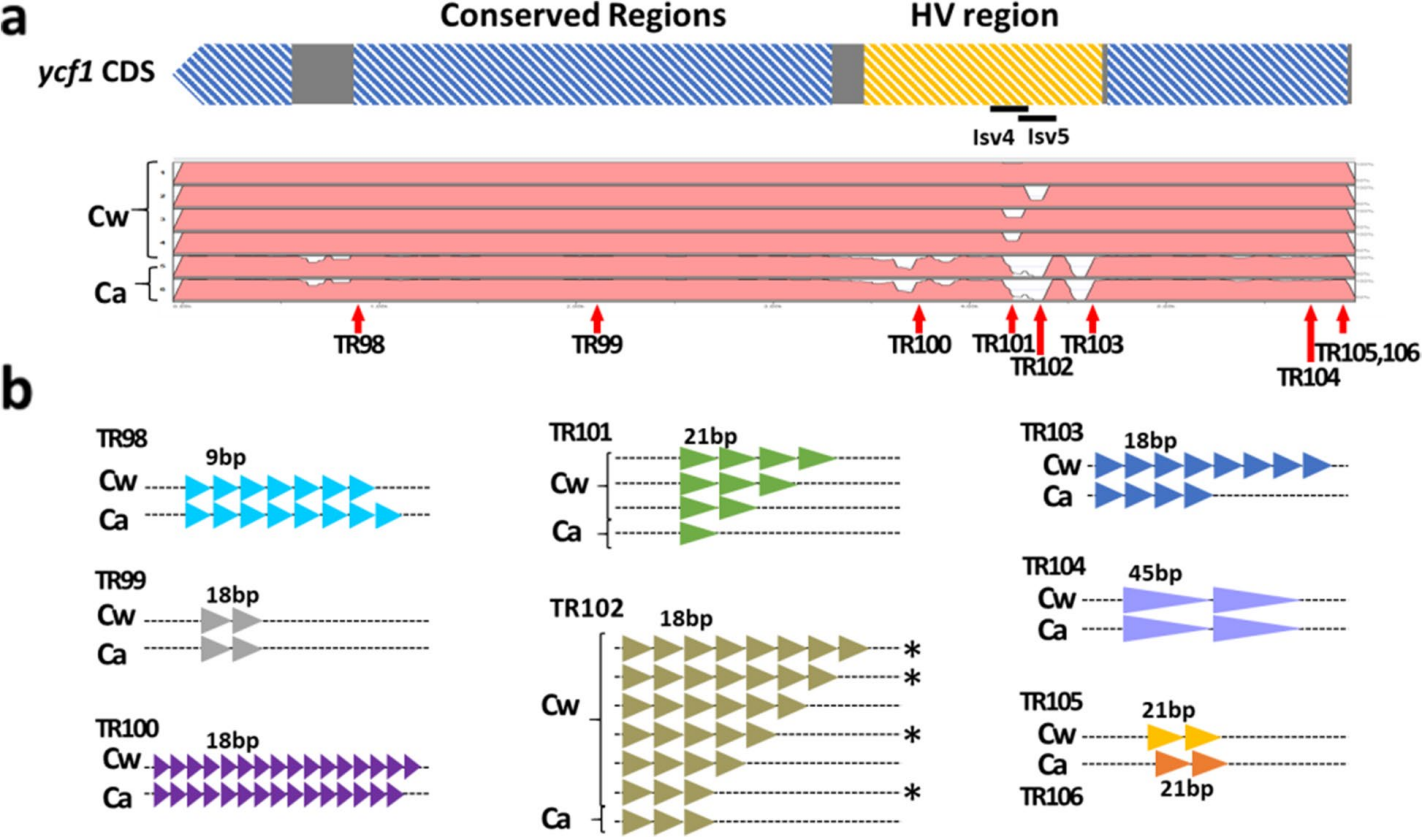
HV sequence derived from TR copy numbers in the *ycf1* gene. **a** *ycf1* gene structure and homology revealed by mVISTA. Gene structure based on sequence variations is indicated: blue, gray, and yellow represent conserved, variable, and HV regions, respectively. Variable regions visualized using the mVISTA program are shown in pink boxes. White regions indicate variable InDel regions. Locations of eight TRs are indicated by red arrows. **b** Polymorphic features of the eight TR units observed in the coding region of the *ycf1* gene. Copy numbers for each TR are denoted by triangle units with different colors. Unexpected genotypes not detected in the sequence but observed in the population are marked with asterisks

We classified 160 *Cynanchum* accessions into 21 haplotypes based on seven plastome markers, four of which showed multiple alleles. Notably, the *accD* and *ycf1* genes, containing three of the five isv markers, showed abundant TR structures within their CDSs (Figs. 5 and 6, S4). The *accD* gene encodes the β-carboxyl transferase subunit of acetyl-CoA carboxylase [22], which is known to be involved in the formation of leaves and chloroplasts in *Nicotiana tabacum* [16]. The *ycf1* gene encodes a subunit of the translocon on the inner chloroplast membrane [13]. Interspecies variations in the coding regions of both genes have been reported many times [15, 27], whereas intraspecific variations in these genes have rarely been reported [3, 4, 10].

The diversity of *accD* and *ycf1* genes is derived from rapid evolution associated with chloroplast competition [32]. In *Medicago truncatula*, *accD* gene function is not affected by differences in internal repeat number because the variable regions are distant from the core binding sites, which have conserved sequences [8]. In Cw and Ca, both genes show HV CNV of diverse TR units, which possibly acts as an additional functional modification despite the lack of frameshift mutations within the open reading frame (ORF) [21].

### Juxtaposition of 45S and 5S nrDNA blocks in one of the 11 chromosomes

To our knowledge, this is the first report of FISH using nrDNA probes for the *Cynanchum* genus. FISH analysis revealed a diploid (2*n* = 2*x* = 22) chromosomal organization of the Cw nuclear genome, similar to those of other plants in the Apocynaceae family identified previously (CCDB-chromosome count database; http://ccdb.tau.ac.il/). One of the 11 Cw chromosomes showed adjacent signals for long tandem arrays of 45S and 5S nrDNA repeats (Fig. 3e, f). We estimated the copy numbers of 45S and 5S nrDNA gene clusters as averaging 554 and 570 copies per haploid genome equivalent, respectively, based on WGS read depth analysis, which is suitable for estimating the copy numbers of major repeat DNA sequences in a genome (Fig. 3e) [20].

A large single-unit nrDNA (L-type) formed from the 5S nrDNA and the 45S nrDNA has been identified in bacteria [18], liverwort, moss [6, 33], yeast [31], and green algae [2]. The L-type nrDNA is hypothesized to be the original ancestral nrDNA organization in the tree of life. Separate loci for the 5S and 45S nrDNA (S-type) blocks are observed in most land plants, indicating that the S-type nrDNA is the ancestral organization in land plants. However, the L-type has been identified in some plants in the Asteraceae [7] and in *Ginkgo biloba* [5], which is predicted to have evolved by eventual reunion of 5S and 45S nrDNA (S-type) independently in those lineages [6, 7]. We observed that the 5S nrDNA array was physically linked to the 45S nrDNA array on Cw chromosome 1, revealing the juxtaposition of 45S and 5S nrDNA blocks. In a cytogenetic study involving 2949 karyotypes from 1791 species, about 33% of karyotypes included juxtaposition of 45S and 5S nrDNA arrays on the same chromosome, mostly on the same chromosome arm [6]. The 11 chromosomes have similar size but only one shows clear characteristics of the juxtaposition of 45S and 5S nrDNA blocks. The defined karyotyping of each the 11 chromosomes is supported by FISH analysis using Cw-specific repeat elements based on genome sequence assembly such as studies of ginseng [35, 36].

### Genetic and morphological diversity among Cw germplasm

**Fig. 7:**
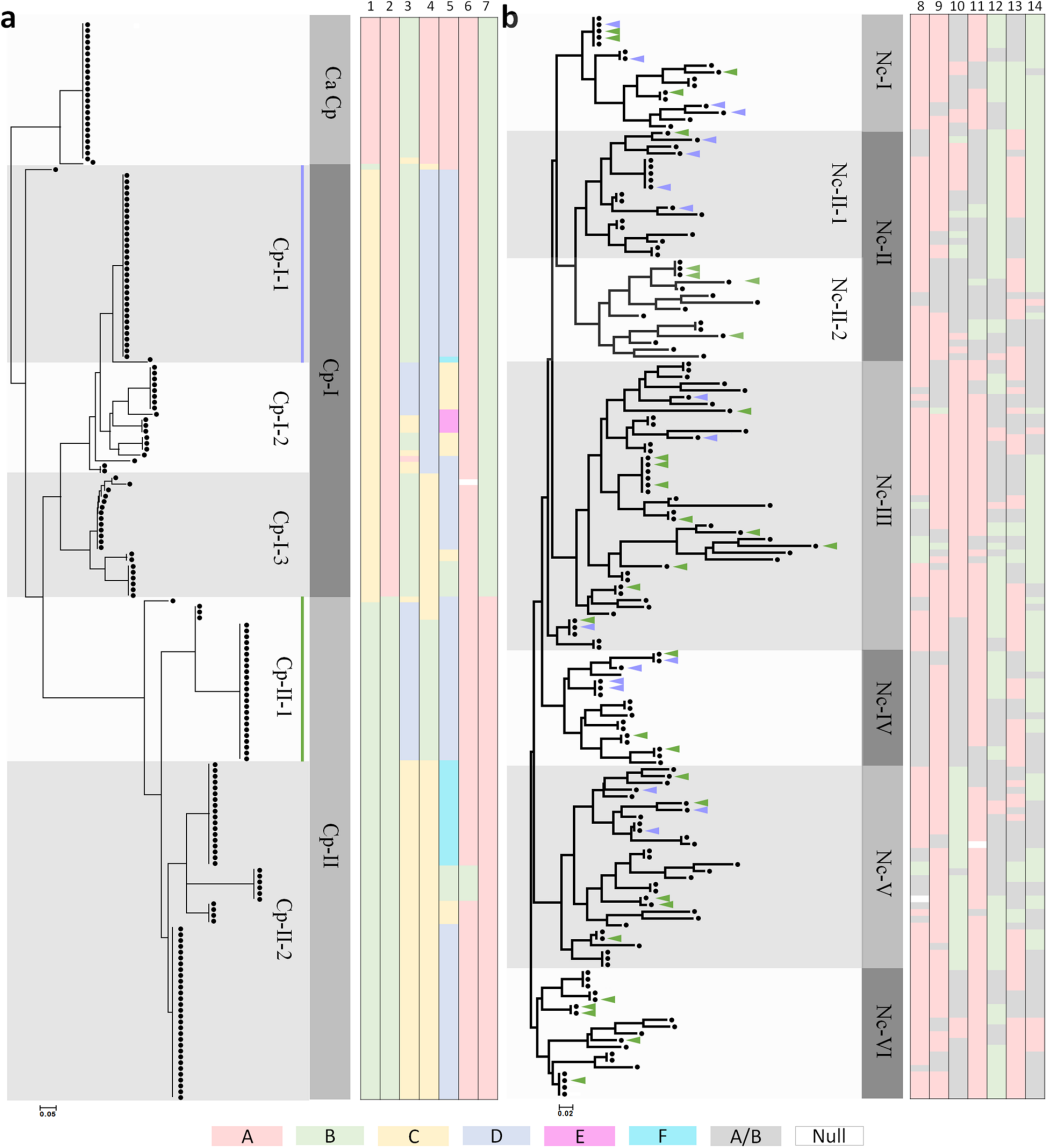
Neighbor-joining tree based on genotyping results of plastome and nuclear genome markers. **a** Chloroplast genome genotype-based neighbor-joining tree of Cw and Ca populations. **b** Nuclear genome genotype-based neighbor-joining tree of the Cw population. Major and minor groups are represented by shaded boxes. Two sets of colored bars represent the genotypes of the chloroplast and nuclear genomes. The seven bars in each set represent genotypes identified with the 14 markers developed from the chloroplast and nuclear genomes, respectively (1: isv1, 2: isv2, 3: isv3, 4: isv4, 5: isv5, 6: isv_cp_kasp1, 7: isv_cp_kasp2, 8: isv_nc_kasp1, 9: isv_nc_kasp2, 10: isv_nc_kasp3, 11: isv_nc_kasp4, 12: isv_nc_kasp5, 13: isv_nc_kasp6, 14: isv_nc_kasp7). Colors indicating each genotype are displayed under the tree. Distribution of Cp-I-1 and Cp-II-1 in the nuclear genome tree is indicated by green and purple triangles

We observed immensely diverse morphological traits of leaves and roots, especially differences in weight. Several Cw plants had leaves similar to those of Ca and also had a vastly different morphology from the typical Cw plants (Figs. 1 and 2). Our unpublished data showed diverse metabolite profiles and anti-inflammatory effects using different Cw collections. We identified considerable genetic diversity within Cw populations through the application of diversity markers in the plastome and nuclear genomes (Fig. 7, Table S8). We therefore believe that the morphological and pharmaceutical diversity of Cw is due to the genetic diversity of this species, which is indigenous to the Korean Peninsula. By contrast, 26 Ca accession showed little variation in plastome markers (Fig. 7, Table S9). Ca was introduced into Korea from China in the form of a few accessions approximately 30 years ago. Therefore, narrow genetic diversity might be expected to exist among Korean accessions of the species (Fig. 7, Table S10).

We identified abundant genetic diversity among Cw accessions from plastome markers and nuclear genome markers. However, the phylogenetic tree based on maternally inherited plastome markers did not coincide with the phylogenetic trees based on the nuclear genome (Fig. 7), suggesting that frequent outcrossing has occurred within the wild Cw germplasm, as for other Apocynaceae family members [23]. The observed heterozygosity of 0.343 and the *k-*mer analysis suggest that the genome possesses high heterozygosity (Fig. 3a). Their immense diversity and heterogeneity suggest their potential as breeding material. Physical map construction and genome-wide association study are needed for efficiently digitalized breeding with the morphological diversity and valuable agricultural traits we have discovered.

Notably, Cw has a special stamen shape, with an anther sac and wing [25], and the stigma head surrounded by a paracorolla. This complex and unusual floral structure is a special feature of the Apocynaceae (Fig. S6). The morphological complexity and small size of Cw flowers make it difficult to identify mature pollen and establish an artificial pollination method. Despite this obstacle to artificial pollination, it will be necessary to breed elite cultivars from the indigenous Cw germplasm to produce uniform agricultural products for functional food ingredients. Good agricultural practice as well as utilization of a favorable cultivar is a primary step for quality control and improvement of functional food species. As highlighted in this study, indigenous plants possess enormous genetic diversity, which can serve as a potential reservoir of useful genetic material. Breeding efforts utilizing this diversity will support the production of diverse elite cultivars yielding specific target-oriented functional foods, promoting sustainable reproduction of the products, which will improve and stimulate the functional food industry under the force of the Nagoya Protocol on Access and Benefit Sharing [1].

## Conclusion

The assembly and comparison of complete plastomes and 45S and 5S nrDNA transcription units revealed that Cw has a relatively small genome size of about 178 Mbp, with 45S and 5S nrDNA in a juxtapositional array on chromosome 1. We identified intraspecies variations through a comparative analysis within the five Cw plastome sequences reported in our previous research, finding that many variations are concentrated in the *accD* and *ycf1* genes. High heterozygosity estimated from *k*-mer analysis and marker application results suggests frequent inbreeding among individuals. The fundamental data and markers developed in this study will be useful for breeding elite cultivars and for further genomic research.

## Materials and methods

### Plant and sequence materials

Cultivated Cw seeds were collected from six local farmers in Geumsan-gun in Chungcheongnam-do, Jecheon city in Chungcheongbuk-do and Andong city in Gyeongsangbuk*-*do. From these seeds, 160 Cw individuals were grown in the field and used for morphological evaluation. Five Cw accessions were used for genome sequencing and genetic diversity analysis (MK182385, MK182386, MK182387, MK182388, NC_029459). All 160 Cw individuals were examined for genetic diversity. Ca seeds for sequencing and marker application were provided by the Rural Development Administration (RDA). Cultivation and evaluation were conducted at the Seoul National University farm, located in Suwon, Gyeonggi-do, Korea. Five Cw and one Ca plastome sequences from our previous research were used for genome analysis [29], and one was from the NCBI (KU900231.1).

### DNA extraction and whole-genome shotgun sequencing

WGSs were produced from five Cw accessions showing different phenotypes using the Illumina platform. Young leaves of each accession were ground using a mortar and pestle with liquid nitrogen. Total genomic DNA was extracted using a QuickGene DNA extraction kit (KURABO Industries LTD) following the manufacturer’s guidelines. The quality and concentration of extracted DNA were measured using a UV spectrophotometer (Thermo Scientific Nanodrop ND-1000). Paired-end library construction and next-generation sequencing (NGS) analysis were conducted using an Illumina MiSeq genome analyzer and HiSeqX genome analyzer by Lab Genomics Inc. (Seongnam, Korea) according to the standard protocol provided by the manufacturer.

### *K-*mer analysis and de novo assembly of plastome and nrDNA sequences

*k-*mer analysis was performed on 160-Gb HiSeqX platform Illumina sequencing data using the JELLYFISH program with a 17-mer option value (http://www.cbcb.umd.edu/software/jellyfish/). After error correction of short reads, *k-*mer frequency analysis revealed a peak in the 17*-*mer depth distribution. The genome size of Cw was estimated from the *k-*mer frequency using the GenomeScope program (http://qb.cshl.edu/genomescope/).

Plastomes and 45S nrDNA sequences of Cw and Ca were assembled using the de novo assembly of low-coverage whole-genome sequencing (dnaLCW) method in the CLC genome assembler program (ver. 4.06 beta, CLC Inc., Aarhus, Denmark). Raw paired-end reads were skimmed with an offset value of 33 and assembled using parameters of overlapping distance ranging from 150 to 500 bp and a window size of 32. Chloroplast-related contigs were extracted from the assembled sequence using MUMmer [17] and the previously reported Cw plastome (NC_029459.1). Extracted contigs were arranged into a single draft sequence and manually curated based on mapping results for NGS reads. The 45S nrDNA and 5S nrDNA contigs were retrieved using the reference sequence of *Panax ginseng*.

### Plastome annotation

Plastomes were annotated using the GeSeq program (https://chlorobox.mpimp-golm.mpg.de) [34] and manually curated using BLAST searches. Circular maps of plastomes were constructed using the OGDRAW program (https://chlorobox.mpimp-golm.mpg.de/OGDraw.html). The 45S nrDNA genome length and coding regions were annotated using the RNAmmer 1.2 program (http://www.cbs.dtu.dk/services/RNAmmer/).

Comparative genome analysis was conducted using MAFFT (www.mafft.cbrc.jp) with five Cw plastomes and two Ca plastomes. After alignment, single-nucleotide polymorphisms (SNPs) and insertions/deletions (InDels) were counted based on inter- or intraspecies polymorphisms. TRs were analyzed using Tandem Repeats Finder (https://tandem.bu.edu) with default parameters. Repeat units were manually verified based on similarity and length. Phylogenetic tree analysis was performed using a power marker program [24] with genotyping results.

### FISH analysis

Chromosome spreads from root mitotic cells of Cw were produced following a method described previously [35]. Pre-labeled oligonucleotide probe (PLOP) FISH probes for 5S and 45S nrDNA loci were utilized in a rapid FISH procedure [36]. Thirty-two microliters of FISH hybridization master mix (50% (v/v) formamide, 10% dextran sulfate and 2× saline-sodium citrate (SSC)) and 25 ng of each PLOP (5S and 45S nrDNA and Arabidopsis-type telomeric repeats) were combined, and then distilled water was added to a total volume of 40 μl. Chromosomal DNA on a glass slide was denatured at 80 °C for 5 min after addition of hybridization mixture.

Hybridization was conducted at room temperature (Waminal et al.) for 1 h and followed by stringent washes in 2× SSC at RT for 5 min, 0.1× SSC at 42 °C for 10 min, and 2× SSC at RT for 5 min. Slides were dehydrated in an ethanol series of 70, 90, and 100% (v/v), air-dried, and counterstained with premixed 4′,6-diamidino-2-phenylindole (DAPI) solution (1 μg/ml DAPI in Vectashield, Vector Laboratories, Burlingame, CA, USA). Images were captured under a model BX53 fluorescence microscope (Olympus, Tokyo, Japan) equipped with a DFC365 FS CCD camera (Leica Microsystems, Wetzlar, Germany) and processed using Cytovision ver. 7.2 (Leica Microsystems). Further image enhancements and karyogram construction were performed using Adobe Photoshop CC (Adobe Systems, San Jose, CA 95110, USA).

### Development of molecular markers

Primers for identifying InDel diversity among Cw plastomes were designed around intraspecific polymorphisms using Primer-BLAST [37] with default parameters. KASP assays for Cw diversity were designed using two intraspecies SNP variation targets and excluding four of the six regions as not suitable for assay design. PCR amplifications were performed as follows: 7 min at 95 °C; 35 cycles of 15–60 s at 95 °C, 15–30 s at 56–60 °C, and 20–30 s at 72 °C; and a final extension for 7 min at 72 °C. Polymorphisms were identified by electrophoresis using a 2% (w/v) agarose gel.

For design of nuclear genome diversity markers, HiSeq data for Cw1 were assembled de novo using QIAGEN CLC Assembler Cell 4.21. MiSeq data for Cw2, Cw3, and Cw4 were aligned to the assembled sequence of Cw1 using Burrows–Wheeler Aligner 0.7.16a with the BWA-MEM algorithm option (http://bio-bwa.sourceforge.net/). Alignment data were sorted using samtools 1.3.1.(http://samtools.sourceforge.net/), and variants were called by samtools Unified Genotyper using the de novo assembled sequence of Cw1 as reference and alignment data for Cw2, Cw3, and Cw4 as inputs. The variants were selected by GATK 3.8 (https://gatk.broadinstitute.org/hc/en-us) with a series of criteria: QUAL ≥30, QD ≥ 2.0, FS ≤ 60.0, MQ ≥ 40.0, BIALLELIC, and DP ≥ 5. The selected variants were filtered using bcftools 1.11 (http://www.htslib.org/doc/bcftools.html) and vcftools 0.1.13 (http://vcftools.sourceforge.net/) with the conditions MIN (FORMAT/DP) > 2 and max-missing 0.66, respectively. Paralogous nuclear plastid and nuclear mitochondrial regions were excluded from marker candidates using BLAST search. The final 10 candidates were visually inspected using CLC assembly viewer and validated using high-resolution melting analysis (HRM). Seven of the 10 candidates were produced for the KASP assay mix. KASP assays of the population were conducted using a LightCycler 480 System (Roche, Applied Science, Indianapolis, IN, USA) according to the manufacturer’s instructions. Analysis of molecular variance was performed using the GenAlex program [30].

## Supplementary Information

**Additional file 1: Supplementary Fig. S1.** Dot plot of population distribution based on morphological measurement data. (a) x and y axis represent average storage root length and thickest root diameter, respectively. (b) x and y axis represent leaf length and width with biggest and oldest leaf of individual. **Supplementary Fig. S2.** Examples of morphological diversity and distribution of Cw individuals in the population. (a) Diversity of length, thickness, diameter and color are presented as a examples from left to the right. (b) Population distribution of Cw according to the morphological diversity. Measured trait ranges and number of Cw are represented with x and y axis, respectively. **Supplementary Fig. S3.** Intra-species single nucleotide polymorphic diversities. Coding regions and inter-, intra-genic regions are presented with navy blocks and light blue dotted lines, respectively. Cw and Ca genotypes are tagged with colored triangles. **Supplementary Fig. S4.** Repetitive motifs found in the inter- and intra-species variation sites of the CDS of the *accD* gene. Inter- and intra-species variation regions in the CDS of the *accD* gene are visualized with schematic diagram according to the mVISTA program (Supplementary Fig. S6). The repeat units found in the center of *accD* gene are represented in a schematic diagram and the shared 13 bp within repeat units are shown in yellow. The unexpected genotype that was not seen in the sequence but observed in the population was marked with *. Genotypes in the sequence but not observe in the population were marked with **. **Supplementary Fig. S5.** Conserved domains regions in *accD* coding site. Inter- and intra-species variation regions in the CDS of the *accD* gene are visualized with the mVISTA program. Similarity of each region compared with Cw1 was indicated with height of pink region. Colored boxes indicate putative Acetyl-coA binding site (red), coA-carboxylation catalytic site (green), carboxy biotin binding site, respectively. **Supplementary Fig. S6.** Complex flower structure of the Cw. (a) Cw flower with calyx removed, 1: anther appendage, 2: pollinarium 2–1: corpusculum, 2–2: caudicle, 2–3: anther sac, 3: petal, 4: calyx, 5: anther wing, (b) Vertically cut Cw flower section, 6: stigma, 7: ovary. **Supplementary Fig. S7.** 160 Cw breeding lines in the field. Overview of Cw breeding field. Cw collections from Korea local farm are cultivated and evaluated in the same condition. Germplasms are continuously maintained as vegetative propagules and seeds. Cw germplasm can be distributed to public through the official contact. **Supplementary Fig. S8.** None-cropped gel picture of Fig. 5. a: isv1, b: isv2, c: isv3, d: isv4, e: isv5. **Supplementary Fig. S9.** Read mapping depth of plastome sequences. Read mapping depth are presented with blue peaks. **Supplementary Table S1.** K-mer analysis result. **Supplementary Table S2.** Sequencing and assembly results of five Cw and Ca collections. **Supplementary Table S3.** Summary of SNPs and InDels found in chloroplast genomes among the two *Cynanchum* species. **Supplementary Table S4.** Analysis of tandem repeats on the complete chloroplast genome sequences. **Supplementary Table S5.** Summary of SNPs and InDels found in 45S nrDNA genomes among the two *Cynanchum* species. **Supplementary Table S6.** Summary of SNPs and InDels found in IGS genomes among the two *Cynanchum* species. **Supplementary data Table S7.** Information of developed molecular markers for identification of intra-species variation of *C. wilfordii.* **Supplementary data Table S8.** Information of KASP markers for identification of intra-species single nucleotide polymorphism variation of *C. wilfordii.* **Supplementary Table S9.** Genotyping result of 165 CW population with seven isv markers. **Table S10.** Analysis of molecular variance result.

## Data Availability

All data analyzed in this study is available upon request. The following data are accessable from NCBI: plastome sequence (MK182385, MK182386, MK182387, MK182388, NC_029459, NC_029460, KU900231.1); 45S nrDNA (MZ156965, MZ156966, MZ156967, MZ156968, MZ156969, MZ156970); 5S nrDNA (MZ246633, MZ246634, MZ246635, MZ246636, MZ246637, MZ246638). SRA (SAMN21583598, SAMN21583599). Raw data for *k*-mer analysis can be provided through the author contact.
