# Supplementary material for: Genome structure and diversity among Cynanchum wilfordii accessions
Source: BMC Plant Biol. 2022 Jan 3;22:4. doi: 10.1186/s12870-021-03390-y (PMC8722063; doi:10.1186/s12870-021-03390-y)
Supplement: Supplementary file 1 — Additional file 1: Supplementary Fig. S1. Dot plot of population distribution based on morphological measurement data. (a) x and y axis represent average storage root length and thickest root diameter, respectively. (b) x and y axis represent leaf length and width with biggest and oldest leaf of individual. Supplementary Fig. S2. Examples of morphological diversity and distribution of Cw individuals in the population. (a) Diversity of length, thickness, diameter and color are presented as a examples from left to the right. (b) Population distribution of Cw according to the morphological diversity. Measured trait ranges and number of Cw are represented with x and y axis, respectively. Supplementary Fig. S3. Intra-species single nucleotide polymorphic diversities. Coding regions and inter-, intra-genic regions are presented with navy blocks and light blue dotted lines, respectively. Cw and Ca genotypes are tagged with colored triangles. Supplementary Fig. S4. Repetitive motifs found in the inter- and intra-species variation sites of the CDS of the accD gene. Inter- and intra-species variation regions in the CDS of the accD gene are visualized with schematic diagram according to the mVISTA program (Supplementary Fig. S6). The repeat units found in the center of accD gene are represented in a schematic diagram and the shared 13 bp within repeat units are shown in yellow. The unexpected genotype that was not seen in the sequence but observed in the population was marked with *. Genotypes in the sequence but not observe in the population were marked with **. Supplementary Fig. S5. Conserved domains regions in accD coding site. Inter- and intra-species variation regions in the CDS of the accD gene are visualized with the mVISTA program. Similarity of each region compared with Cw1 was indicated with height of pink region. Colored boxes indicate putative Acetyl-coA binding site (red), coA-carboxylation catalytic site (green), carboxy biotin binding site, respectively. Supple [file 12870_2021_3390_MOESM1_ESM.docx]

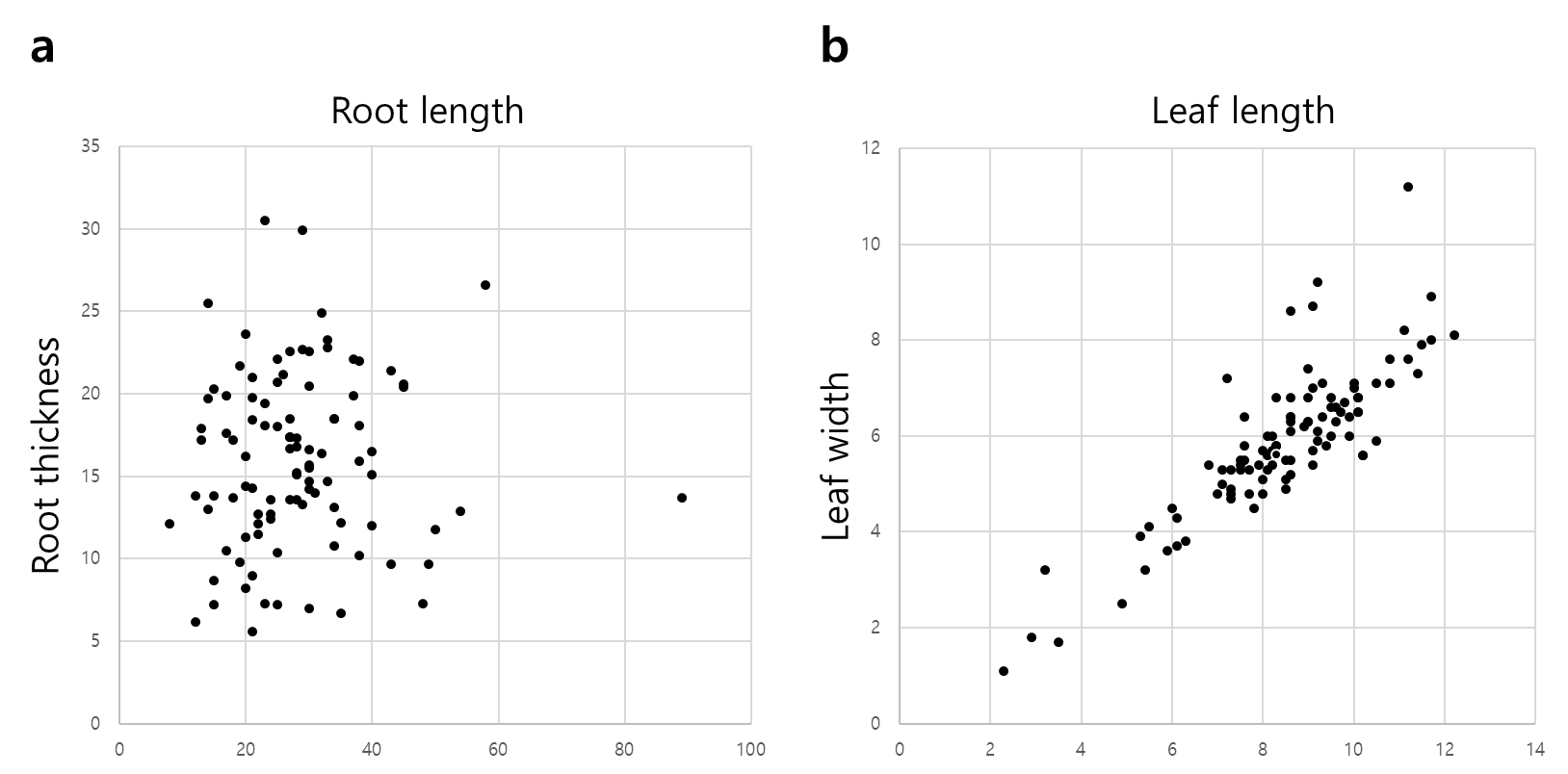


**Supplementary Fig. S1 Dot plot of population distribution based on morphological measurement data**

(a) x and y axis represent average storage root length and thickest root diameter, respectively. (b) x and y axis represent leaf length and width with biggest and oldest leaf of individual.

**
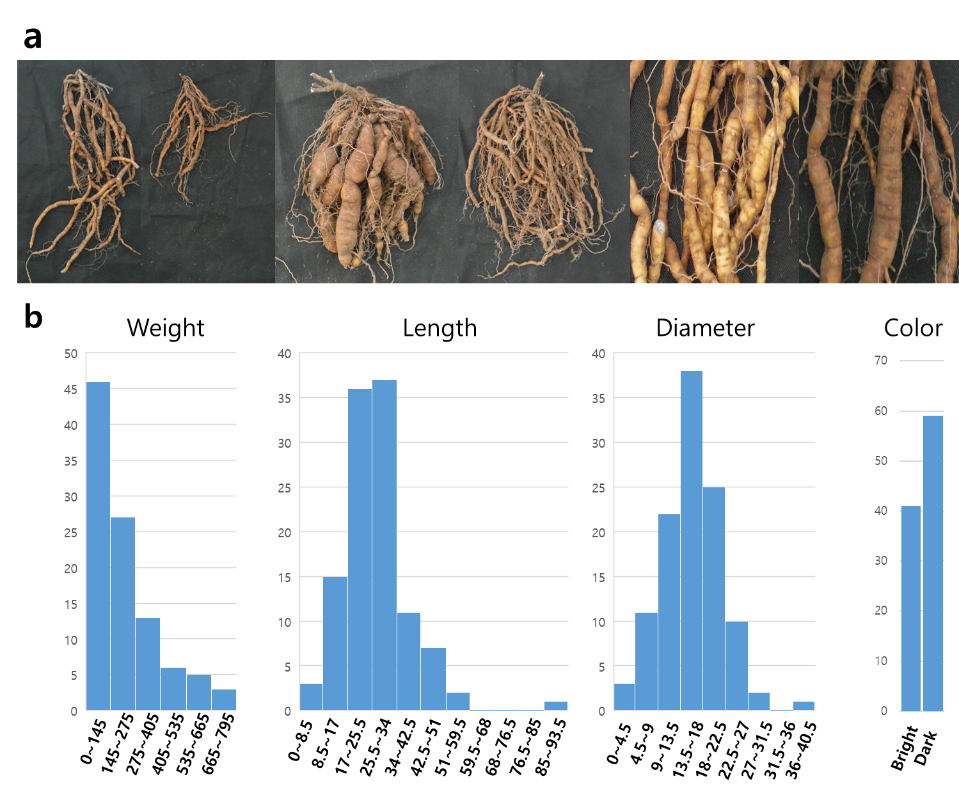
**

**Supplementary Fig. S2 Examples of morphological diversity and distribution of Cw individuals in the population.**

(a) Diversity of length, thickness, diameter and color are presented as a examples from left to the right. (b) Population distribution of Cw according to the morphological diversity. Measured trait ranges and number of Cw are represented with x and y axis, respectively.

**
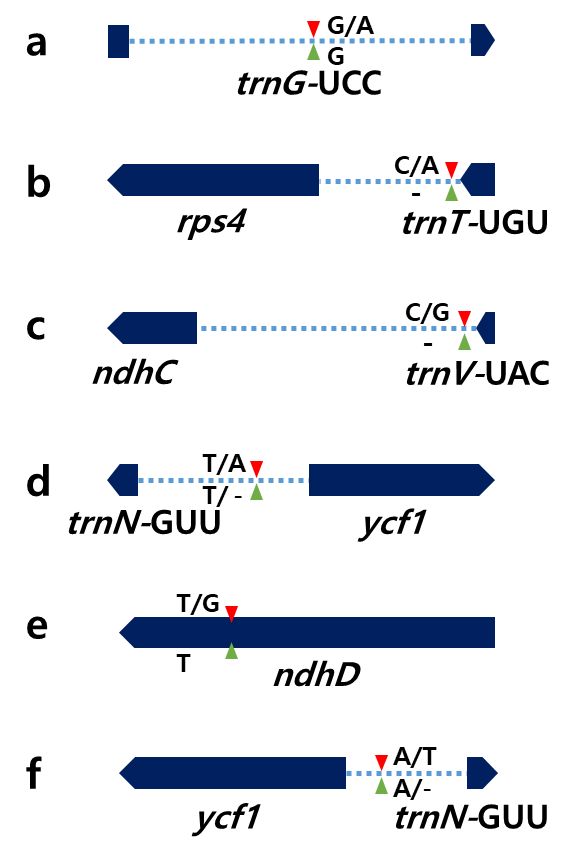
**

**Supplementary Fig. S3 Intra-species single nucleotide polymorphic diversities**

Coding regions and inter-, intra-genic regions are presented with navy blocks and light blue dotted lines, respectively. Cw and Ca genotypes are tagged with colored triangles.

**
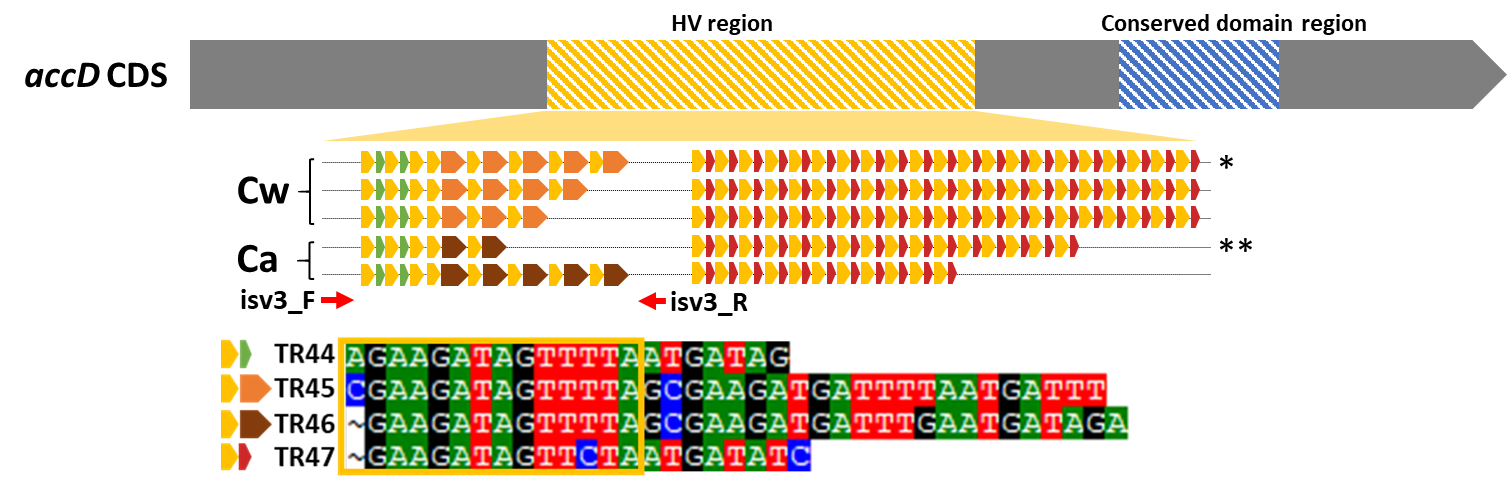
**

**Supplementary Fig. S4 Repetitive motifs found in the inter- and intra-species variation sites of the CDS of the *accD* gene.** Inter- and intra-species variation regions in the CDS of the *accD* gene are visualized with schematic diagram according to the mVISTA program (Supplementary Fig. S6). The repeat units found in the center of *accD* gene are represented in a schematic diagram and the shared 13 bp within repeat units are shown in yellow. The unexpected genotype that was not seen in the sequence but observed in the population was marked with *. Genotypes in the sequence but not observe in the population were marked with **.

**
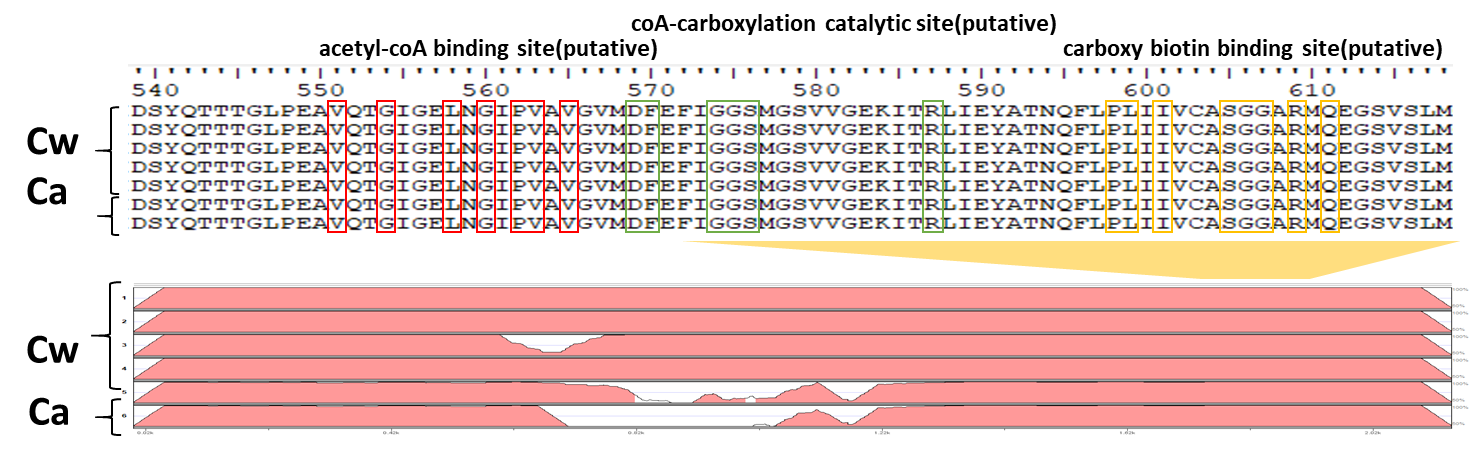
**

**Supplementary Fig. S5 Conserved domains regions in *accD* coding site.** Inter- and intra-species variation regions in the CDS of the *accD* gene are visualized with the mVISTA program. Similarity of each region compared with Cw1 was indicated with height of pink region. Colored boxes indicate putative Acetyl-coA binding site (red), coA-carboxylation catalytic site (green), carboxy biotin binding site, respectively.


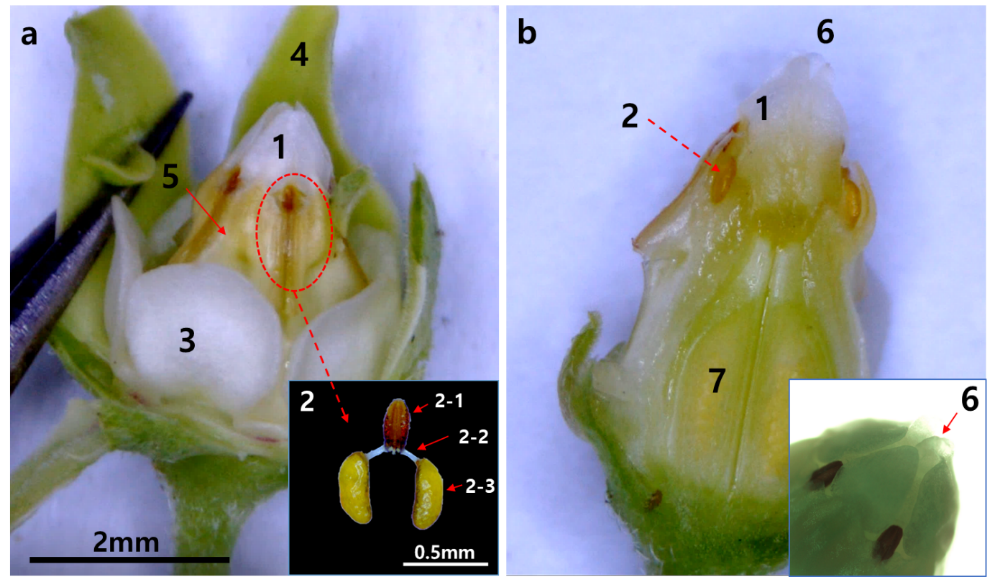


Supplementary Fig. S6 Complex flower structure of the Cw. (a) Cw flower with calyx removed, 1: anther appendage, 2: pollinarium 2-1: corpusculum, 2-2: caudicle, 2-3: anther sac, 3: petal, 4: calyx, 5: anther wing, (b) Vertically cut Cw flower section, 6: stigma, 7: ovary


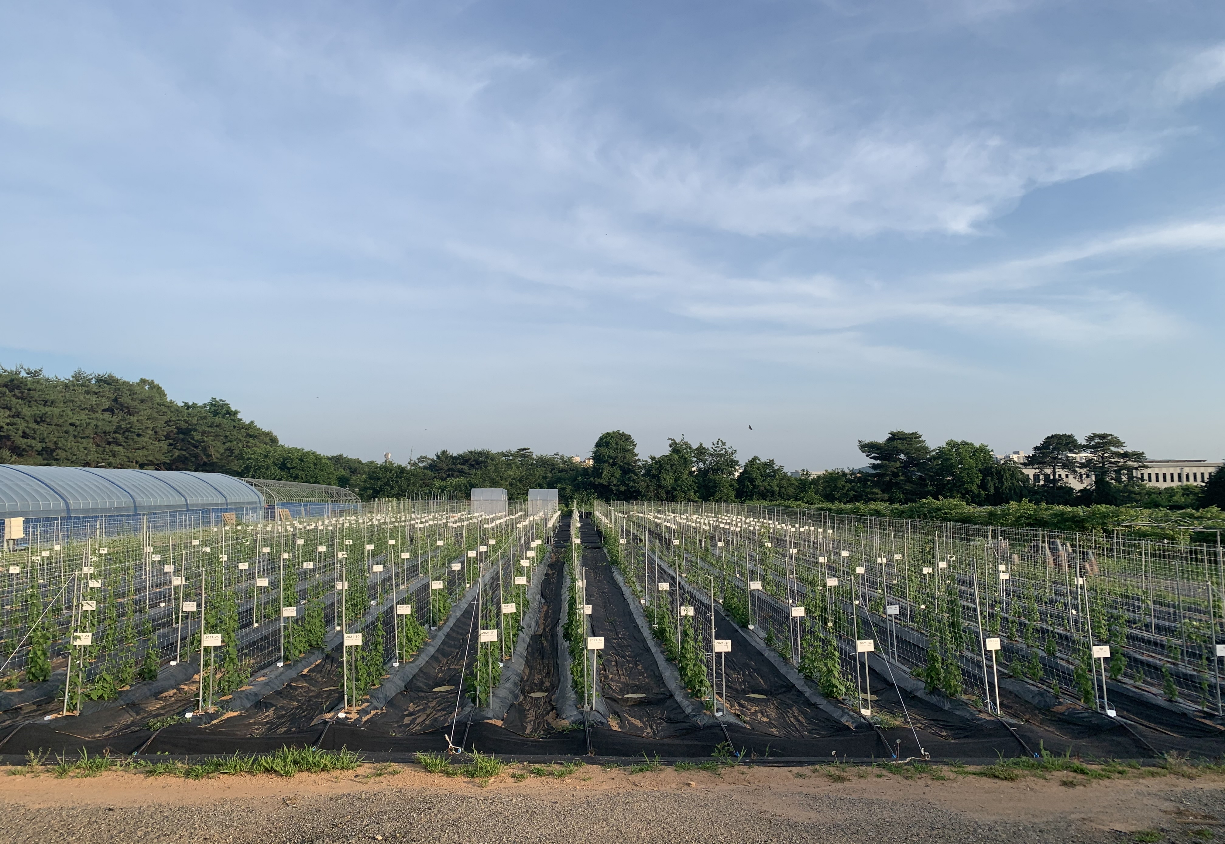


Supplementary Fig. S7. 160 Cw breeding lines in the field. Overview of Cw breeding field. Cw collections from Korea local farm are cultivated and evaluated in the same condition. Germplasms are continuously maintained as vegetative propagules and seeds. Cw germplasm can be distributed to public through the official contact.


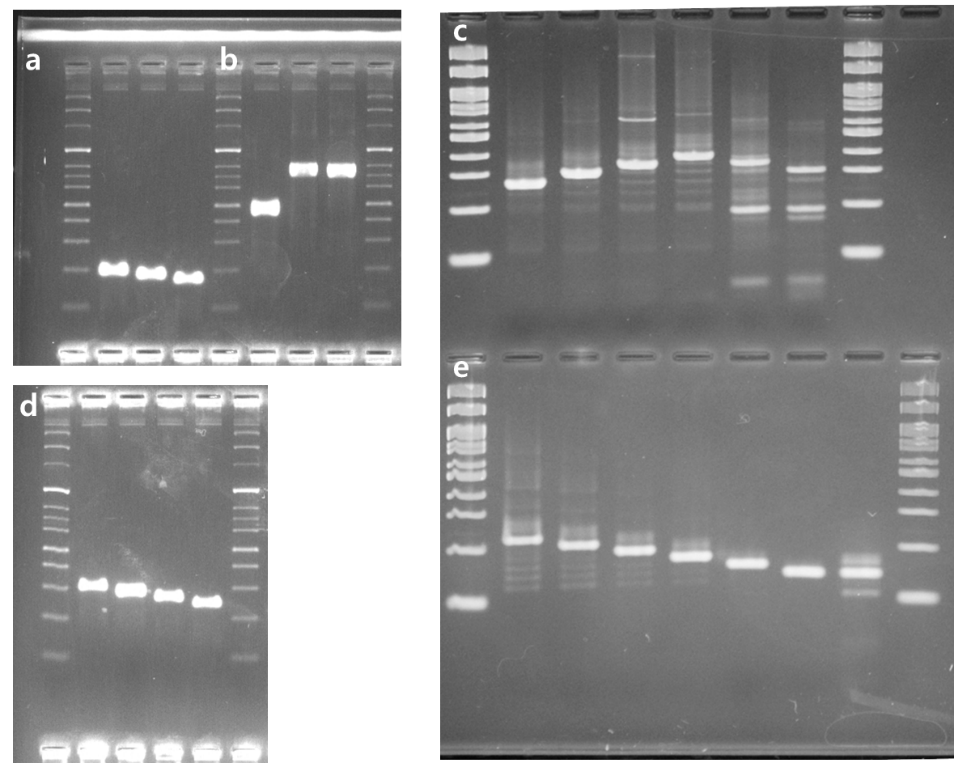


Supplementary Fig. S8. None-cropped gel picture of Figure 5. a: isv1, b: isv2, c: isv3, d: isv4, e: isv5


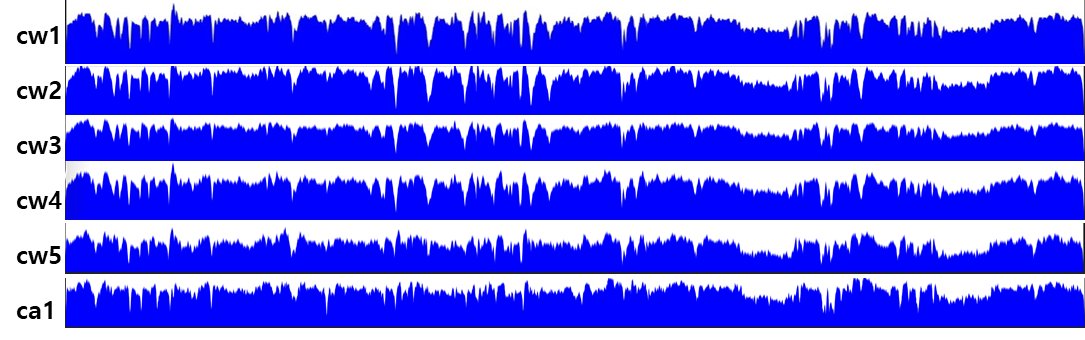


Supplementary Fig. S9. Read mapping depth of plastome sequences. Read mapping depth are presented with blue peaks.

**Supplementary Table S1** K-mer analysis result

|  | 17mer a |
| --- | --- |
| Data amount (Gb) | 160 |
| Estimated depth at peak  (k-mer coverage depth) | 159 |
| Genome heterozygosity (%) | 0.576 |
| Estimated Genome Size (bp) | 178,610,210 |

**Supplementary Table S2. Sequencing and assembly results of five Cw and Ca collections**

|  | Feature | Cw1 | Cw2 | Cw3 | Cw4 | Cw5 | Ca1 |
| --- | --- | --- | --- | --- | --- | --- | --- |
| Collection site | | Jecheon | Geumsan | Geumsan | Geumsan | Eumseong | Eumseong |
| Genebank acc. number | | MK182385 | MK182386 | MK182387 | MK182388 | NC_029459 | NC_029460 |
| Sequencing reads (bp) | | 63,921,394,821 | 1,036,194,961 | 804,480,225 | 1,058,297,665 | 724,363,954 | 801,590,026 |
| Chloroplast genome | Size (bp) | 160,864 | 160,865 | 160,829 | 160,808 | 161,241 | 160,840 |
|  | LSC (bp) | 91,641 | 91,642 | 91,642 | 91,606 | 91,995 | 91,973 |
|  | SSC (bp) | 19,907 | 19,907 | 19,871 | 19,886 | 19,930 | 19,667 |
|  | IR (bp) | 24,658 | 24,658 | 24,658 | 24,658 | 24,658 | 24,600 |
| Copy No. ^z^ | | 683 | 674 | 514 | 432 | 482 | 799 |
| 45S nrDNA | Gene bank acc.number | MZ156965 | MZ156966 | MZ156967 | MZ156968 | MZ156969 | MZ156970 |
|  | Size (bp) | 5,842 | 5,842 | 5,842 | 5,842 | 5,842 | 5,842 |
|  | 18S (bp) | 1,807 | 1,807 | 1,807 | 1,807 | 1,807 | 1,807 |
|  | 5.8S (bp) | 164 | 164 | 164 | 164 | 164 | 164 |
|  | 26S (bp) | 3,392 | 3,392 | 3,392 | 3,392 | 3,392 | 3,392 |
|  | Copy No. ^y^ | 474 | 383 | 604 | 511 | 256 | 266 |
| 5S nrDNA | Gene bank acc.number | MZ246633 | MZ246634 | MZ246635 | MZ246636 | MZ246637 | MZ246638 |
|  | Size (bp) | 444 | 444 | 444 | 444 | 444 | 448 |
|  | 5S | 115 | 115 | 115 | 115 | 115 | 115 |
|  | IGS | 329 | 329 | 329 | 329 | 329 | 333 |
|  | Copy No. ^y^ | 429 | 385 | 545 | 767 | 233 | 209 |

**Abbreviation**: Cw1~4: Four sequenced *C. wilfordii* individual, Cw5: Park *et al.* 2016, Ca1: Jang *et al.* 2016

Note: C = Estimated copy numbers of chloroplast and nrDNA, D = Mapping depth of raw data on the chloroplast and nrDNA genome, G = Calculated haploid genome size with *k-*mer analysis, R = Total read bases. C = D*G/R.; ^z^Chloroplast copy number is calculated with diploid genome size. ^y^nrDNA copy number is calculated with haploid genome size.

**Supplementary Table S3** Summary of SNPs and InDels found in chloroplast genomes among the two *Cynanchum* species

|  | Cw1 | Cw2 | Cw3 | Cw4 | Cw5 | Ca1 | Ca2 |
| --- | --- | --- | --- | --- | --- | --- | --- |
| Cw1 |  | 1 | 2 | 5 | 8 | 253 | 251 |
| Cw2 | 2 |  | 1 | 4 | 7 | 252 | 250 |
| Cw3 | 1 | 3 |  | 5 | 8 | 252 | 250 |
| Cw4 | 0 | 2 | 1 |  | 9 | 253 | 252 |
| Cw5 | 3 | 5 | 4 | 3 |  | 252 | 250 |
| Ca1 | 970 | 973 | 972 | 967 | 973 |  | 4 |
| Ca2 | 923 | 924 | 925 | 924 | 926 | 11 |  |

**Note:** The lower triangle shows the total nucleotide substitutions, while the upper triangle indicates the number of InDels.

**Abbreviations:** Cw1 : *C. wilfordii* (MK182385), Cw2 : *C. wilfordii* (MK182386)*,* Cw3 : *C. wilfordii* (MK182387)*,* Cw4 : *C. wilfordii* (MK182388)*,* Cw5 : *C. wilfordii* (NC_029459), Ca1 : *C. auriculatum* (NC_029460), Ca2 : *C. auriculatum* (KU900231.1)

**Supplementary Table S4** Analysis of tandem repeats on the complete chloroplast genome sequences

| Marker | No. | TR unit sequence | length | location | | Copy number | |
| --- | --- | --- | --- | --- | --- | --- | --- |
|  |  |  |  |  |  | cw | ca |
|  | TR1 | AATATTTTAGAATAT | 15 | *trnH-GUG - psbA* | intergenic | 3 | 0 |
|  | TR2 | ATACGTATTTCTATAC | 16 | *psbA - trnK-UUU* | intergenic | 1 | 2 |
|  | TR3 | ATTGCAATTACA | 12 | *matK* | CDS | 2 | 2 |
|  | TR4 | ATTAGAAAATATATGTATA | 19 | *trnK-UUU - rps16* | intergenic | 2 | 1 |
|  | TR5 | AATAAATTAAAC | 12 | *trnK-UUU - rps16* | intergenic | 2 | 2 |
|  | TR6 | TGAGCTAACAAA | 12 | *trnK-UUU - rps16* | intergenic | 2 | 2 |
|  | TR7 | TTGTTCTATAAA | 12 | *rps16 - trnQ-UUG* | intergenic | 2 | 2 |
|  | TR8 | ATAAAGTATAATT | 13 | *trnQ-UUG - psbK* | intergenic | 2 | 1 |
|  | TR9 | ATTAATTATTAT | 12 | *trnQ-UUG - psbK* | intergenic | 0 | 2 |
|  | TR10 | TATATATAGTA | 11 | *trnQ-UUG - psbK* | intergenic | 0 | 3 |
|  | TR11 | TAATTATAATTAG | 13 | *trnQ-UUG - psbK* | intergenic | 1 | 2 |
|  | TR12 | TAATTAATATTTTAATAATTT | 21 | *trnQ-UUG - psbK* | intergenic | 1 | 2 |
|  | TR13 | TTAAAATTTTAATTAA | 16 | *trnQ-UUG - psbK* | intergenic | 2 | 0 |
|  | TR14 | CTTAGTATTATATAAA | 16 | *trnS-GCU - trnG-UCC* | intergenic | 1 | 2 |
|  | TR15 | TTTTATTTAATTC | 13 | *atpH - atpI* | intergenic | 2 | 2 |
|  | TR16 | TCTTGTCATGTATATGAGTAT | 21 | *rps2 - rpoC2* | intergenic | 2 | 2 |
|  | TR17 | AATTCTTAATAATTAAAG | 18 | *rpoC1* | intron | 1 | 2 |
|  | TR18 | ATAACCATATG | 11 | *rpoC1* | intron | 2 | 2 |
|  | TR19 | AAGTTTAAGTTAAGTTATAGGATA | 24 | *rpoB - trnC-GCA* | intergenic | 2 | 3 |
|  | TR20 | TATTATTATTATGATATTGC | 20 | *rpoB - trnC-GCA* | intergenic | 2 | 2 |
|  | TR21 | TTTGTATTTCGTAAAG | 16 | *petN - psbM* | intergenic | 2 | 2 |
|  | TR22 | ATACATATATAATAT | 15 | *petN - psbM* | intergenic | 2 | 2 |
|  | TR23 | AGAGAGGTCCTGAACCGCTAGACGATGGGGGCCCATTTGTCCAACCGTCATGATACTATAATC | 63 | *trnE-UUU - trnT-GGU* | intergenic | 2 | 2 |
|  | TR24 | TATCTCTATATCTATA | 16 | *trnE-UUU - trnT-GGU* | intergenic | 2 | 1 |
|  | TR25 | ATCTCTATATCTATATA | 17 | *trnE-UUC - trnT-GGU* | intergenic | 1 | 2 |
|  | TR26 | TGTATATATATGTATATATACTGTATATATAC | 32 | *trnE-UUU - trnT-GGU* | intergenic | 2 | 0 |
|  | TR27 | TATATATTAATA | 12 | *psbZ - trnG-GCC* | intergenic | 2 | 0 |
|  | TR28 | ATATATTAATATA | 13 | *psbZ - trnG-GCC* | intergenic | 0 | 2 |
|  | TR29 | TTATACAGAATGGCAAAGGG | 20 | *trnfM-CAU - rps14* | intergenic | 2 | 2 |
|  | TR30 | TTATTTAATTCTTTA | 15 | *psaA - ycf3* | intergenic | 2 | 2 |
|  | TR31 | AATAGAGTACTTAG | 14 | *psaA - ycf3* | intergenic | 2 | 2 |
|  | TR32 | CAAATTATATACTTATATAC | 20 | *psaA - ycf3* | intergenic | 0 | 2 |
| Isv1 | TR33 | TAATTATTTAATT | 13 | *ycf3* | intron | 2~3 | 1 |
|  | TR34 | ATGATACAAAGAAGA | 15 | *ycf3 - trnS-GGA* | intergenic | 2 | 2 |
|  | TR35 | ATTAAAAATTATTA[-/A] | 15 | *rps4 - trnT-UGU* | intergenic | 2 | 3 |
|  | TR36 | TCAATTTTATGTGC | 14 | *trnT-UGU - trnL-UAA* | intergenic | 2 | 1 |
|  | TR37 | TCTTATTTTCACCTCTGT | 18 | *ndhC - trnV-UAC* | intergenic | 2 | 2 |
|  | TR38 | AATTATTATTACTTATTACTATCTATTACT | 30 | *ndhC - trnV-UAC* | intergenic | 2 | 2 |
|  | TR39 | ATTTATTTA[-/A]TT | 11 | *ndhC - trnV-UAC* | intergenic | 4 | 4 |
|  | TR40 | ATATTAATAGTATTAG | 16 | *ndhC - trnV-UAC* | intergenic | 2 | 0 |
|  | TR41 | ATATTGCTTAAATTA | 15 | *trnV-UAC - trnM-CAU* | intergenic | 3 | 3 |
|  | TR42 | TAATAATTTTAATAATTTATAATATATTTTAATATTAA | 38 | *rbcL - accD* | intergenic | 2 | 1 |
|  | TR43 | ATTTAATAATAAATTTTAT | 19 | *rbcL - accD* | intergenic | 2 | 2 |
|  | TR44 | AGAAGATAGTTTTAATGATAG | 21 | *accD* | CDS | 2 | 2 |
| Isv3 | TR45 | CGAAGATAGTTTTAGCGAAGATGATTTTAATGATTT | 36 | *accD* | CDS | 3~4 | 0 |
| Isv3 | TR46 | GAAGATAGTTTTAGCGAAGATGATTTGAATGATAGA | 36 | *accD* | CDS | 0 | 2~5 |
|  | TR47 | GAAGATAGTTCTAATGATATC | 21 | *accD* | CDS | 21 | 11~16 |
|  | TR48 | GATGAGATACTAAAATAAAA | 20 | *petA - psbJ* | intergenic | 2 | 2 |
|  | TR49 | TCTATTAGTATAGA | 14 | *petA - psbJ* | intergenic | 2 | 2 |
|  | TR50 | TTTGTTAGTACCGTCTATAATG | 22 | *psbE - petL* | intergenic | 1 | 2 |
|  | TR51 | ATTTAATCCACAG | 13 | *petG - trnW-CCA* | intergenic | 2 | 2 |
|  | TR52 | ATCATATAATAAACATAT | 18 | *trnP-UGG - psaJ* | intergenic | 2 | 1 |
|  | TR53 | AACATATAATAACATAT | 17 | *trnP-UGG - psaJ* | intergenic | 0 | 2 |
|  | TR54 | ATATAATATAAAATATAATATAAAATATAATATATATAATATATAATAATAGTTAA | 56 | *trnP-UGG - psaJ* | intergenic | 2 | 0 |
|  | TR55 | ATTTTAGTTATTGAG | 15 | *psaJ - rpl33* | intergenic | 2 | 1 |
|  | TR56 | ATTTACTATGACTTTA | 16 | *psaJ - rpl33* | intergenic | 3 | 3 |
|  | TR57 | AGAACC | 6 | *rps18* | CDS | 5 | 5 |
|  | TR58 | TTTATTTATTTATTATTTAA | 20 | *rpl20 - rps12* | intergenic | 2 | 1 |
|  | TR59 | TTTATTATTTATTATTTAATTTTATTATTTATTATTTAATATTTAA | 46 | *rpl20 - rps12* | intergenic | 0 | 2 |
|  | TR60 | AAATTAAATAAGTAAATTAA | 20 | *rpl20 - rps12* | intergenic | 0 | 6 |
|  | TR61 | TAAAATTAAA | 10 | *rpl20 - rps12* | intergenic | 5 | 0 |
|  | TR62 | TCACCTGCTACAAGATCA | 18 | *clpP* | CDS | 6 | 6 |
|  | TR63 | TTCTATCGAGTTTGTTCATTAACAG | 25 | *clpP* | intron | 1 | 3 |
|  | TR64 | TTCTAATTGATAATAGTAA | 19 | *clpP* | intron | 1 | 2 |
|  | TR65 | TAATTGCTAATGATAATAG | 19 | *clpP* | intron | 2 | 1 |
|  | TR66 | ATTTAATAGTAT | 12 | *petD* | intron | 2 | 2 |
|  | TR67 | ATTTTTTTATATTTTATCTGG | 21 | *petD* | intron | 0 | 2 |
|  | TR68 | ATCCTTCGAATCCTTGTT | 18 | *infA* | CDS | 4 | 3 |
|  | TR69 | TTTTATGAATA | 11 | *rps8 - rpl14* | intergenic | 1 | 3 |
|  | TR70 | AATTAATTAATTAATATTA | 19 | *rpl16* | intron | 1 | 3 |
|  | TR71 | ACTAATTAAGATTAAGATAT | 20 | *rpl16* | intron | 2 | 1 |
|  | TR72 | TTTAATTATGTTATAT | 16 | *rpl16* | intron | 2 | 2 |
|  | TR73 | TATATTTAATAATTTAA | 17 | *rpl22 - rps19* | intergenic | 1 | 2 |
|  | TR74 | AATAAGAATGCTAGTTCTTACTGTTCATAAATTACGTATTATG | 43 | *rpl23 - trnI-CAU* | intergenic | 2 | 2 |
|  | TR75 | ACGATATTGCTGCTAGTG | 18 | *ycf2* | CDS | 2 | 2 |
|  | TR76 | GAAGAGGGGGAGGGAGAA | 18 | *ycf2* | CDS | 2 | 2 |
|  | TR77 | GAGGAAGAGGCTG[A/C]GCTTCGA | 21 | *ycf2* | CDS | 4 | 3 |
|  | TR78 | TAAAAAATAAAAAAAA | 16 | *trnA-UGC - 23S ribosomal RNA* | intergenic | 2 | 1 |
|  | TR79 | CATTGTTCAAATCTTTGACAACACGAAAAAA | 31 | *4.5S ribosomal RNA 5S ribosomal RNA* | intergenic | 2 | 2 |
|  | TR80 | TCATATAATGATCCACAGAAAT | 22 | *trnN-GUU - ycf1* | intergenic | 2 | 2 |
|  | TR81 | TTTCATATAATG | 12 | *trnN-GUU - ycf1* | intergenic | 12 | 7~15 |
|  | TR82 | AAAGTTTTTCAATTATTGAAA | 21 | *ycf1* | CDS | 6 | 3 |
|  | TR83 | TTTTACGATTCATTTTACAATGTA | 24 | *ycf1* | CDS | 2 | 2 |
|  | TR84 | TAATATTACTTCTTACTTAATTT | 23 | *ycf1* | CDS | 3 | 0 |
|  | TR85 | AATAAAAACCTAAAATCCCT | 20 | *ndhF* | CDS | 2 | 2 |
|  | TR86 | TACTT[T/A]ACTTA | 11 | *ndhF - rpl32* | intergenic | 7 | 5 |
|  | TR87 | TTTTTTATTATAAA | 14 | *ndhF - rpl32* | intergenic | 1 | 2 |
|  | TR88 | TTATTATTAAGAT | 13 | *ndhF - rpl32* | intergenic | 2 | 2 |
|  | TR89 | ATTCCTATGGTACAACAGATTCTACAGATATCAACTTCAATGAGAAAGAATATC | 54 | *ndhF - rpl32* | intergenic | 2 | 2 |
|  | TR90 | ATTAAGTAATTTAAGT | 16 | *rpl32 - trnL-UAG* | intergenic | 5 | 1 |
|  | TR91 | TTAAGTATTAAGTAAT | 16 | *rpl32 - trnL-UAG* | intergenic | 1 | 2 |
|  | TR92 | TATATAGAAAATAGTATAGAATAAAAATATAGAAT | 35 | *rpl32 - trnL-UAG* | intergenic | 2 | 0 |
|  | TR93 | ATAGAATTAGATAAAAT | 17 | *rpl32 - trnL-UAG* | intergenic | 0 | 2 |
|  | TR94 | TATATATAAATATAAATATAACTCT | 25 | *ndhG - ndhI* | intergenic | 2 | 0 |
|  | TR95 | TTAATATTAATTAA | 14 | *ndhG - ndhI* | intergenic | 3 | 3 |
|  | TR96 | CTTTTACTATATCAAAAC | 18 | *ndhH* | CDS | 2 | 2 |
|  | TR97 | ATTTTTATGTGTTAT | 15 | *rps15 - ycf1* | intergenic | 1 | 2 |
|  | TR98 | TCTTTTTTA | 9 | *ycf1* | CDS | 7 | 8 |
|  | TR99 | TAAAGTAAATTATATTTT | 18 | *ycf1* | CDS | 2 | 2 |
|  | TR100 | TTTTGATTTTCATTT[A/G]CA | 18 | *ycf1* | CDS | 16 | 15 |
| Isv4 | TR101 | TTGGTAATCGGGATCTCCAAA | 21 | *ycf1* | CDS | 2~4 | 1 |
| Isv5 | TR102 | CTTCTATCGACTTGTCAG | 18 | *ycf1* | CDS | 4~6 | 3 |
|  | TR103 | ATCCTTGGTTTTT[C/T]TATT | 18 | *ycf1* | CDS | 8 | 4 |
|  | TR104 | AAGGTATCCATTTTAGCTTTGGCCCAAA[G/A]AACTTCCTAGTTAGAG | 45 | *ycf1* | CDS | 2 | 2 |
|  | TR105 | TAAAACGAAAACTTAAATAAT | 21 | *ycf1* | CDS | 2 | 0 |
|  | TR106 | TGAAAAAATTAAAAGGAAAAT | 21 | *ycf1* | CDS | 0 | 2 |
|  | TR107 | CATTATATGAAA | 12 | *trnN-GUU - ycf1* | intergenic | 12 | 7~15 |
|  | TR108 | ATTTCTGTGGATCATTATATGA | 22 | *trnN-GUU - ycf1* | intergenic | 2 | 2 |
|  | TR109 | TTTTTTCGTGTTGTCAAAGATTTGAACAATG | 31 | *4.5S ribosomal RNA - 5S ribosomal RNA* | intergenic | 2 | 2 |
|  | TR110 | TTTTTTTTATTTTTTA | 16 | *ndhB* | intron | 2 | 1 |
|  | TR111 | TCGAAGC[G/T]CAGCCTCTTCCTC | 21 | *ycf2* | CDS | 4 | 3 |
|  | TR112 | TTCTCCCTCCCCCTCTTC | 18 | *ycf2* | CDS | 2 | 2 |
|  | TR113 | CACTAGCAGCAATATCGT | 18 | *ycf2* | CDS | 2 | 2 |
|  | TR114 | CATAATACGTAATTTATGAACAGTAAGAACTAGCATTCTTATT | 43 | *rpl23 - trnI-CAU* | intergenic | 2 | 2 |
|  | TR115 | TTAAATTATTAAATATA | 17 | *rpl22 - rps19* | intergenic | 1 | 2 |

**Note:** The sequence [N_1_ / N_2_] represents a different sequence between Cw and Ca respetively. The forward slash indicates Cw and the back indicates the base sequence of Ca ; Units with identity less than 70% were ignored.

**Supplementary Table S5** Summary of SNPs and InDels found in 45S nrDNA genomes among the two *Cynanchum* species

|  | Cw1 | Cw2 | Cw3 | Cw4 | Cw5 | Ca1 |
| --- | --- | --- | --- | --- | --- | --- |
| Cw1 |  | 0 | 0 | 0 | 0 | 0 |
| Cw2 | 1 |  | 0 | 0 | 0 | 0 |
| Cw3 | 2 | 3 |  | 0 | 0 | 0 |
| Cw4 | 0 | 1 | 2 |  | 0 | 0 |
| Cw5 | 1 | 0 | 3 | 1 |  | 0 |
| Ca1 | 15 | 16 | 13 | 15 | 16 |  |

**Note:** The lower triangle shows the total nucleotide substitutions, while the upper triangle indicates the number of InDels.

**Abbreviations:** Cw1 : *C. wilfordii* (MK182385), Cw2 : *C. wilfordii* (MK182386)*,* Cw3 : *C. wilfordii* (MK182387)*,* Cw4 : *C. wilfordii* (MK182388)*,* Cw5 : *C. wilfordii* (NC_029459), Ca1 : *C. auriculatum* (NC_029460), Ca2 : *C. auriculatum* (KU900231.1)

**Supplementary Table S6** Summary of SNPs and InDels found in IGS genomes among the two *Cynanchum* species

|  | Cw1 | Cw2 | Cw3 | Cw4 | Cw5 | Ca1 |
| --- | --- | --- | --- | --- | --- | --- |
| Cw1 |  | 0 | 0 | 0 | 0 | 2 |
| Cw2 | 0 |  | 0 | 0 | 0 | 2 |
| Cw3 | 0 | 0 |  | 0 | 0 | 2 |
| Cw4 | 0 | 0 | 0 |  | 0 | 2 |
| Cw5 | 0 | 0 | 0 | 0 |  | 2 |
| Ca1 | 13 | 13 | 13 | 13 | 13 |  |

**Note:** The lower triangle shows the total nucleotide substitutions, while the upper triangle indicates the number of InDels.

**Abbreviations:** Cw1 : *C. wilfordii* (MK182385), Cw2 : *C. wilfordii* (MK182386)*,* Cw3 : *C. wilfordii* (MK182387)*,* Cw4 : *C. wilfordii* (MK182388)*,* Cw5 : *C. wilfordii* (NC_029459), Ca1 : *C. auriculatum* (NC_029460), Ca2 : *C. auriculatum* (KU900231.1)

**Supplementary data Table S7** Information of developed molecular markers for identification of intra-species variation of *C. wilfordii*

| **Primer** | **Sequence (5ʹ to 3ʹ)** | **Location** | | **Melting temp.**  **(°C)** | **Product size (bp)** | | **Allele no.** | | **Repeat size(bp)** |
| --- | --- | --- | --- | --- | --- | --- | --- | --- | --- |
|  |  |  |  |  | Cw | Ca | **Cw** | **Ca** |  |
| isv1* | F: CCTTGGTGCCGCGTTTTAAT | *ycf3* | intron | 58.0 | 181 | 207 | 2 | 1 | 13 |
|  | R: CGTTATTCTTCTGACGGTGGGA |  |  |  |  |  |  |  |  |
| isv2* | F: TGATCGAATTGACTAGTTTCCTTTG | *ndhC-atpE* | intergenic | 58.0 | 449,  839 | 819 | 2 | 1 | (390) |
|  | R: GGACCTGTCGTGCTTGTGTA |  |  |  |  |  |  |  |  |
| isv3* | F: TAATGGCGAAACGGAGGGTT | *accD* | CDS | 58.0 | 239 | 311 | 3 | 2 | 36 |
|  | R: GAATTAACTCACTATCTTGCATC |  |  |  |  |  |  |  |  |
| isv4 | F: CGCTGATTAAGTCGCTTGGC | *ycf1* | CDS | 58.0 | 268 | 268 | 3 | 1 | 21 |
|  | R: CGGATTTACACTCTCCTTTTTGGA |  |  |  |  |  |  |  |  |
| isv5 | F: AGGAGAGTGTAAATCCGATTTATA | *ycf1* | CDS | 58.0 | 158 | 140 | 5 | 1 | 18 |
|  | R: AGTGGGCCCTATCGCTTAAC |  |  |  |  |  |  |  |  |

**Abbreviations:** Cw : *C. wilfordii*, Ca : *C. auriculatum*

**Note:** isv2 is non-repeat region and amplicon size difference is indicated in the bracket

* =Cw_i_6,7,8 (Park *et al.* 2019)

**Supplementary data Table S8** Information of KASP markers for identification of intra-species single nucleotide polymorphism variation of *C. wilfordii*

| **Primer** | **Sequence (5ʹ to 3ʹ)** | **Gene name** | **Location** | **Genotype** | | **Melting temp.(°C)** |
| --- | --- | --- | --- | --- | --- | --- |
|  |  |  |  | **CW** | **CA** |  |
| isv_cp_kasp1 | F1: ACTCTGGTTTACTAGAGGCATCG | *trnG*-UCC | Intron | T | T | 58.0 |
|  | F2: GACTCTGGTTTACTAGAGGCATCA |  |  | G | - |  |
|  | R : AAAGGGATTTTGTTTCCACCGAGCTAAAA |  |  |  |  |  |
| isv_cp_kasp2 | F1: TCTTGGCAGGGACAACTTATGATC | *ndhD* | CDS | G | G | 58.0 |
|  | F2: TTCTTGGCAGGGACAACTTATGATA |  |  | A | - |  |
|  | R : CATTTCGTCAAGATAAAGAAGGCGTGTT |  |  |  |  |  |
| isv_nc_kasp1 | F1: AGTGCCCAGAGCAAAGACAGC | TOR 1-like | CDS | G | G | 58.0 |
|  | F2: ATAGTGCCCAGAGCAAAGACAGA |  |  | T |  |  |
|  | R: GACATGTACTGAGACAACATTTTGTAGGTT |  |  |  |  |  |
| isv_nc_kasp2 | F1: CTGATGCTGATATCTTCGTTACC | Ahcy-like | CDS | G | G | 58.0 |
|  | F2: CTCTGATGCTGATATCTTCGTTACT |  |  | A |  |  |
|  | R: CAACCATGATGATGTCTTTGTTACCAGTT |  |  |  |  |  |
| isv_nc_kasp3 | F1: GATTATTGAGATGCGTGATGAGGAC | EMB2765-like | CDS | G |  | 58.0 |
|  | F2: AGATTATTGAGATGCGTGATGAGGAA |  |  | T | T |  |
|  | R: ACTCATCTCGCTTTATCCTTCCAGTAAA |  |  |  |  |  |
| isv_nc_kasp4 | F1: TGCTACAGACATTGCACGCAGAA | AIR carboxylase-like | CDS | T |  | 58.0 |
|  | F2: GCTACAGACATTGCACGCAGAG |  |  | C | C |  |
|  | R: AAATATACCTGCACCTTCCAATGAGCTAA |  |  |  |  |  |
| isv_nc_kasp5 | F1: GGTCTTTACATATCCTCATCATAACAG | Uncharacterized | Uncharacterized | G |  | 58.0 |
|  | F2: CTGGTCTTTACATATCCTCATCATAACAA |  |  | A | A |  |
|  | R: GCCCCATATAAGATGAACCTAAAGATGTA |  |  |  |  |  |
| isv_nc_kasp 6 | F1: ATGTGAATGTGAGAATACTTAATTACCTC | LTO1-like | CDS | G |  | 58.0 |
|  | F2: ATGTGAATGTGAGAATACTTAATTACCTG |  |  | C | C |  |
|  | R: TTGAAGGATTCCCTACATGGGTAATCAAT |  |  |  |  |  |
| isv_nc_kasp7 | F1: ACCAAAGGAGGAAATCAATGAACTAGT | Uncharacterized | uncharacterized | G |  | 58.0 |
|  | F2: CCAAAGGAGGAAATCAATGAACTAGC |  |  | C | C |  |
|  | R: GCAAGAGGAGGCACTTCAATCCTTA |  |  |  |  |  |

**Supplementary Table S9** Genotyping result of 165 CW population with seven isv markers.

| acc. No. | CP marker | | | | | | | Nuclear marker | | | | | | |
| --- | --- | --- | --- | --- | --- | --- | --- | --- | --- | --- | --- | --- | --- | --- |
|  | ISV1 | ISV2 | ISV3 | ISV4 | ISV5 | isv_cp_kasp1 | Isv_cp_kasp2 | isv_nc_kasp1 | isv_nc_kasp2 | isv_nc_kasp3 | isv_nc_kasp4 | isv_nc_kasp5 | isv_nc_kasp6 | isv_nc_kasp7 |
| cw1 | C | A | B | D | D | A | B | A/A | A/A | A/A | A/B | B/B | B/B | A/B |
| cw2 | C | A | C | D | C | A | B | A/A | A/A | A/B | A/A | B/B | A/A | A/B |
| cw3 | B | B | D | B | D | A | A | A/A | A/A | A/B | B/B | B/B | A/B | A/B |
| cw4 | C | A | B | C | C | A | B | A/B | A/A | A/A | A/A | B/B | B/B | B/B |
| cw5 | C | A | B | C | D | A | B | A/A | A/A | A/B | A/B | A/B | A/B | A/B |
| cw6 | C | A | B | C | D | A | B | A/B | A/B | A/B | A/B | A/B | A/A | A/A |
| cw7 | C | A | B | D | D | A | B | A/A | A/A | A/A | B/B | A/B | A/B | A/B |
| cw8 | B | B | D | C | D | A | A | A/A | A/A | B/B | A/A | A/B | A/B | A/B |
| cw9 | C | A | B | D | D | A | B | B/B | A/A | A/A | A/B | B/B | B/B | B/B |
| cw10 | B | B | D | C | D | A | A | A/A | A/A | A/B | A/A | A/B | A/A | A/B |
| cw11 | C | A | D | D | C | A | B | A/A | A/A | A/A | A/A | A/B | A/B | B/B |
| cw12 | C | A | D | D | C | A | B | A/A | A/A | A/B | A/A | B/B | A/B | B/B |
| cw13 | B | B | C | C | F | A | A | A/B | A/B | A/B | A/A | B/B | A/B | B/B |
| cw14 | C | A | C | D | D | A | B | A/A | A/A | A/B | A/B | B/B | B/B | B/B |
| cw15 | C | A | B | D | D | A | B | A/B | A/B | A/B | A/A | B/B | A/B | B/B |
| cw16 | B | B | C | C | F | A | A | A/A | A/A | A/B | A/A | B/B | A/A | B/B |
| cw17 | B | B | D | B | D | A | A | A/B | A/A | A/B | A/A | A/B | A/A | B/B |
| cw18 | C | B | C | C | D | A | A | A/A | A/A | A/A | A/A | B/B | A/B | B/B |
| cw19 | B | B | C | C | D | A | A | A/A | A/B | A/A | A/B | B/B | A/B | A/B |
| cw20 | C | A | B | C | B | A | B | A/A | A/A | A/B | A/A | B/B | A/A | A/B |
| cw21 | C | A | B | C | B | A | B | A/A | A/B | A/B | A/A | B/B | A/A | A/B |
| cw22 | B | B | C | C | D | A | A | A/A | A/A | A/B | A/A | A/B | A/A | B/B |
| cw23 | B | B | D | B | D | A | A | A/A | A/A | A/B | A/A | A/B | A/B | B/B |
| cw24 | B | B | C | C | D | A | A | B/B | A/B | A/A | A/A | A/B | B/B | B/B |
| cw25 | B | B | C | C | D | A | A | A/B | A/B | A/A | A/A | A/B | A/B | A/B |
| cw26 | B | B | C | C | D | A | A | A/A | A/A | A/B | A/B | B/B | A/A | B/B |
| cw27 | B | B | C | C | D | A | A | A/A | A/A | A/B | A/A | A/B | B/B | B/B |
| cw28 | C | A | B | D | D | A | B | A/B | A/A | A/A | A/A | A/B | A/B | B/B |
| cw29 | B | B | C | C | D | A | A | A/B | A/A | A/A | A/A | A/B | A/A | B/B |
| cw30 | C | A | B | D | D | A | B | A/A | A/A | A/B | A/A | B/B | A/B | B/B |
| cw31 | C | A | B | D | C | A | B | A/B | A/A | A/B | A/A | A/B | A/B | A/B |
| cw32 | C | A | B | D | D | A | B | A/A | A/A | A/A | A/A | A/B | A/B | B/B |
| cw33 | B | A | B | C | A | A | B | A/A | A/B | B/B | A/A | B/B | A/A | B/B |
| cw34 | C | A | C | D | E | A | B | A/A | A/A | A/A | A/B | A/B | A/A | A/B |
| cw35 | C | A | C | D | E | A | B | A/A | A/A | A/B | A/A | B/B | B/B | B/B |
| cw36 | B | B | C | C | F | A | A | A/B | A/A | A/B | A/A | B/B | A/A | B/B |
| cw37 | B | B | C | C | F | A | A | A/A | A/A | B/B | A/A | A/B | A/B | A/B |
| cw38 | B | B | C | C | F | A | A | A/A | A/B | A/A | A/A | A/B | A/B | A/B |
| cw39 | C | A | B | D | D | A | B | A/A | A/A | A/B | A/A | B/B | A/B | A/B |
| cw40 | C | A | B | D | D | A | B | A/A | A/A | A/B | A/A | B/B | A/B | A/B |
| cw41 | B | B | C | C | D | A | A | A/B | A/A | A/B | A/B | A/B | A/A | A/B |
| cw42 | C | A | B | C | D | A | B | A/B | A/A | A/B | A/A | A/B | A/B | B/B |
| cw43 | C | A | B | C | D | A | B | B/B | A/A | B/B | A/A | A/B | B/B | B/B |
| cw44 | C | A | B | C | D | A | B | A/B | A/A | B/B | A/A | A/B | A/B | B/B |
| cw45 | B | B | D | B | D | A | A | A/B | A/A | B/B | A/A | B/B | B/B | A/B |
| cw46 | B | B | D | B | D | A | A | A/A | A/A | A/B | A/B | B/B | A/B | B/B |
| cw47 | B | B | D | B | D | A | A | A/A | A/A | B/B | A/B | A/B | A/B | A/B |
| cw48 | B | B | C | C | C | A | A | A/A | A/A | B/B | A/A | B/B | A/A | A/B |
| cw49 | C | A | B | C | D | A | B | A/A | A/A | A/A | A/A | A/B | A/A | A/B |
| cw50 | C | A | B | C | D | A | B | A/A | A/A | A/A | A/A | A/B | A/B | A/B |
| cw51 | B | B | C | C | D | A | A | A/A | A/A | A/B | A/B | B/B | B/B | B/B |
| cw52 | C | A | D | D | C | A | B | A/A | A/A | A/A | A/A | A/B | A/A | A/A |
| cw53 | B | B | D | B | D | A | A | A/A | A/A | A/A | A/A | A/A | A/B | A/A |
| cw54 | B | B | D | B | D | A | A | A/A | A/A | A/A | A/A | A/B | A/B | B/B |
| cw55 | B | B | C | C | D | A | A | A/A | A/A | B/B | A/B | B/B | A/B | B/B |
| cw56 | C | A | B | D | D | A | B | A/A | A/A | B/B | A/A | A/A | A/A | A/B |
| cw57 | B | B | C | C | D | A | A | A/A | A/A | A/B | A/B | A/B | A/A | B/B |
| cw58 | C | A | B | D | D | A | B | A/A | A/A | A/B | A/A | B/B | A/A | B/B |
| cw59 | B | B | C | C | B | B | A | A/A | A/A | A/A | A/A | B/B | A/A | A/B |
| cw60 | C | A | B | D | D | A | B | A/A | A/B | A/B | A/B | B/B | A/A | B/B |
| cw61 | B | B | D | B | D | A | A | A/B | A/A | A/A | A/A | A/B | A/B | B/B |
| cw62 | B | B | D | B | D | A | A | A/B | A/A | A/B | A/A | B/B | A/B | A/B |
| cw63 | C | A | B | D | D | A | B | A/B | A/A | A/B | A/A | A/B | A/A | A/B |
| cw64 | B | B | D | B | D | A | A | A/A | A/A | A/A | A/B | B/B | A/A | B/B |
| cw65 | C | A | B | D | D | A | B | A/A | A/A | A/B | A/A | A/B | A/A | A/A |
| cw66 | C | A | B | D | D | A | B | A/A | A/A | A/B | A/A | B/B | B/B | B/B |
| cw67 | C | A | B | C | D | A | B | A/B | A/A | B/B | A/A | A/B | B/B | B/B |
| cw68 | C | A | A | D | D | A | B | A/A | A/B | B/B | A/A | A/B | A/B | A/B |
| cw69 | B | B | C | C | D | A | A | A/A | A/A | A/A | A/A | A/B | A/A | A/B |
| cw70 | C | A | B | D | D | A | B | A/B | A/A | A/B | A/B | B/B | A/A | B/B |
| cw71 | B | B | C | C | D | A | A | A/A | A/A | A/B | A/B | B/B | A/B | B/B |
| cw72 | C | A | B | C | B | A | B | A/A | A/B | A/B | A/A | B/B | A/A | A/B |
| cw73 | B | B | C | C | D | A | A | A/A | A/A | B/B | A/A | A/B | A/A | B/B |
| cw74 | B | B | C | C | D | A | A | B/B | A/B | A/A | A/B | A/B | A/B | B/B |
| cw75 | C | A | B | D | D | A | B | A/A | A/A | A/A | A/A | A/B | A/B | B/B |
| cw76 | C | A | B | D | C | A | B | A/B | A/A | A/B | A/A | A/B | A/B | B/B |
| cw77 | B | B | C | C | F | A | A | A/A | A/B | A/B | A/A | A/B | B/B | B/B |
| cw78 | B | B | D | B | D | A | A | A/A | A/A | B/B | A/B | B/B | B/B | A/B |
| cw79 | B | B | C | C | C | A | A | A/A | A/A | A/B | A/A | B/B | A/A | A/B |
| cw80 | B | B | D | B | D | A | A | A/A | A/B | A/B | A/B | A/B | A/A | A/B |
| cw81 | C | A | B | D | D | A | B | A/A | A/B | A/B | B/B | A/B | A/A | B/B |
| cw82 | B | B | D | B | D | A | A | A/A | A/B | B/B | A/B | B/B | A/B | B/B |
| cw83 | C | A | B | D | D | A | B | A/B | A/A | B/B | A/A | B/B | A/B | B/B |
| cw84 | B | B | D | B | D | A | A | A/A | A/A | A/A | A/A | A/B | A/B | A/B |
| cw85 | C | A | B | D | D | A | B | A/B | A/A | A/B | A/B | B/B | A/A | A/B |
| cw86 | B | B | D | B | D | A | A | A/A | A/A | A/B | B/B | B/B | A/B | A/B |
| cw87 | C | A | B | D | D | A | B | A/A | A/A | A/A | A/A | B/B | A/A | A/B |
| cw88 | C | A | B | D | D | A | B | -/- | A/A | B/B | A/A | B/B | A/B | B/B |
| cw89 | C | A | B | D | D | A | B | A/A | A/A | A/B | A/A | A/B | A/B | A/B |
| cw90 | C | A | B | C | C | A | B | A/A | A/A | A/B | A/A | A/B | A/A | A/B |
| cw91 | B | B | D | C | D | A | A | A/A | A/A | B/B | A/A | A/B | A/A | A/B |
| cw92 | C | A | D | D | C | A | B | A/A | A/A | A/A | A/B | B/B | A/A | B/B |
| cw93 | C | A | C | D | D | A | B | A/A | A/A | A/B | A/A | A/B | A/B | A/B |
| cw94 | B | B | C | C | F | A | A | A/B | A/A | A/B | A/A | B/B | A/B | B/B |
| cw95 | B | B | C | C | D | A | A | A/A | A/A | A/B | A/B | B/B | A/A | B/B |
| cw96 | C | A | B | C | B | A | B | A/A | A/A | A/B | A/A | B/B | A/A | B/B |
| cw97 | B | B | D | B | D | A | A | A/A | A/B | A/A | A/A | B/B | A/A | A/A |
| cw98 | B | B | C | C | D | A | A | A/B | A/B | A/B | A/B | A/B | A/B | A/B |
| cw99 | B | B | C | C | D | A | A | A/B | A/A | A/B | A/A | B/B | A/A | B/B |
| cw100 | C | A | B | D | D | A | B | A/B | A/B | A/A | A/A | B/B | B/B | B/B |
| cw101 | C | A | B | D | D | A | B | A/A | A/A | B/B | A/A | B/B | A/A | B/B |
| cw102 | C | A | D | D | E | A | B | A/A | A/A | A/B | A/B | A/A | A/A | A/B |
| cw103 | C | A | C | D | E | A | B | A/A | A/A | A/A | A/B | B/B | A/A | B/B |
| cw104 | B | B | C | C | F | A | A | A/A | A/A | B/B | A/A | A/A | A/B | A/B |
| cw105 | B | B | C | C | F | A | A | A/A | A/A | A/B | A/A | B/B | A/B | B/B |
| cw106 | B | B | C | C | F | A | A | A/A | A/A | A/A | A/A | A/A | A/A | A/B |
| cw107 | B | B | C | C | D | A | A | A/B | A/A | A/A | A/A | B/B | A/A | A/B |
| cw108 | B | B | C | C | D | A | A | A/B | A/A | A/A | A/A | B/B | A/A | B/B |
| cw109 | C | A | B | C | D | - | B | A/A | A/A | A/A | A/A | B/B | A/B | B/B |
| cw110 | C | A | B | C | D | A | B | A/A | A/A | B/B | A/A | B/B | A/B | A/B |
| cw111 | C | A | B | C | D | A | B | A/A | A/A | B/B | A/A | B/B | A/A | B/B |
| cw112 | B | B | C | C | C | A | A | A/A | A/B | A/B | A/B | A/B | A/A | B/B |
| cw113 | C | A | B | C | D | A | B | A/A | A/A | A/A | A/B | B/B | A/A | B/B |
| cw114 | C | A | D | D | C | A | B | A/A | A/B | A/A | A/A | A/B | A/A | A/A |
| cw115 | B | B | D | B | D | A | A | A/A | A/B | B/B | -/- | A/B | A/B | A/B |
| cw116 | B | B | C | C | D | A | A | A/A | A/A | B/B | A/B | A/B | A/A | B/B |
| cw117 | C | A | B | D | D | A | B | A/A | A/A | B/B | A/B | A/B | A/B | B/B |
| cw118 | B | B | C | C | B | B | A | A/A | A/A | A/B | A/A | A/B | A/A | B/B |
| cw119 | B | B | D | B | D | A | A | A/A | A/A | B/B | B/B | B/B | A/A | B/B |
| cw120 | B | B | D | B | D | A | A | A/A | A/A | B/B | A/A | B/B | A/A | A/B |
| cw121 | B | B | D | B | D | A | A | A/B | A/B | A/B | A/A | B/B | A/A | A/B |
| cw122 | C | A | B | D | D | A | B | A/A | B/B | A/A | A/A | A/B | A/A | B/B |
| cw123 | B | B | D | B | D | A | A | A/A | A/A | A/B | A/A | B/B | A/B | B/B |
| cw124 | C | A | B | D | D | A | B | A/A | A/A | A/B | A/A | B/B | A/A | A/B |
| cw125 | C | A | B | D | D | A | B | A/A | A/B | A/B | A/B | B/B | A/A | B/B |
| cw126 | B | B | C | C | D | A | A | A/A | A/B | A/A | A/A | A/B | A/A | A/B |
| cw127 | C | A | B | D | D | A | B | B/B | B/B | A/A | A/B | B/B | A/B | B/B |
| cw128 | C | A | B | C | D | A | B | A/B | A/A | A/B | A/A | A/B | A/A | B/B |
| cw129 | B | B | D | B | D | A | A | A/A | A/B | A/A | A/A | B/B | A/B | B/B |
| cw130 | B | B | C | C | B | B | A | A/A | A/B | A/A | A/A | A/B | A/A | A/B |
| cw131 | B | B | C | C | B | B | A | A/A | A/A | A/B | A/A | B/B | A/A | A/A |
| cw132 | B | B | C | C | B | B | A | A/A | A/A | A/B | A/A | A/B | A/A | A/B |
| cw133 | B | B | C | C | B | B | A | A/B | A/A | B/B | A/A | A/B | A/B | A/B |
| cw134 | C | A | B | D | D | A | B | A/A | A/A | A/B | A/A | B/B | A/B | B/B |
| cw135 | C | A | D | D | C | A | B | A/A | A/A | A/A | A/A | A/B | B/B | B/B |
| cw136 | C | A | D | D | C | A | B | A/A | A/A | A/A | A/A | B/B | A/A | B/B |
| cw137 | C | A | D | D | C | A | B | A/A | A/A | A/A | A/A | A/B | A/B | B/B |
| cw138 | B | B | C | C | F | A | A | A/B | A/A | A/A | A/B | B/B | A/B | B/B |
| cw139 | B | B | C | C | F | A | A | A/A | A/A | A/B | B/B | B/B | A/A | B/B |
| cw140 | B | B | C | C | F | A | A | A/B | A/A | B/B | A/B | B/B | A/A | B/B |
| cw141 | B | B | C | C | F | A | A | A/B | A/A | A/B | A/A | B/B | A/A | B/B |
| cw142 | B | B | C | C | F | A | A | A/A | A/A | A/A | A/B | B/B | A/A | B/B |
| cw143 | C | A | B | D | F | A | B | A/B | A/A | B/B | A/A | A/B | A/B | A/B |
| cw144 | B | B | C | C | D | A | A | A/B | A/A | A/B | A/A | A/B | A/A | A/B |
| cw145 | B | B | C | C | D | A | A | A/A | A/A | A/A | A/B | B/B | B/B | B/B |
| cw146 | C | A | B | C | B | A | B | A/A | A/B | A/A | A/A | B/B | A/A | A/B |
| cw147 | C | A | B | C | B | A | B | A/B | A/A | A/B | A/B | B/B | A/A | A/B |
| cw148 | B | B | C | C | D | A | A | B/B | A/A | A/A | A/A | A/A | A/A | B/B |
| cw149 | B | B | C | C | D | A | A | A/B | A/B | A/A | A/B | B/B | A/A | A/B |
| cw150 | B | B | C | C | D | A | A | A/A | A/B | A/B | A/B | B/B | A/A | B/B |
| cw151 | B | B | C | C | D | A | A | A/A | A/B | A/B | A/B | B/B | A/B | B/B |
| cw152 | B | B | C | C | D | A | A | A/A | A/A | B/B | A/B | B/B | A/B | B/B |
| cw153 | B | B | C | C | D | A | A | A/B | A/A | A/A | A/B | B/B | A/A | B/B |
| cw154 | C | A | B | D | C | A | B | B/B | A/A | A/B | A/A | A/B | B/B | B/B |
| cw155 | C | A | B | D | D | A | B | A/A | A/A | A/A | A/A | A/B | A/B | B/B |
| cw156 | B | B | C | C | F | A | A | A/A | A/A | B/B | A/A | A/B | A/B | B/B |
| cw157 | B | B | C | C | F | A | A | A/A | A/A | A/B | A/A | A/B | A/B | B/B |
| cw158 | B | B | C | C | F | A | A | A/A | A/B | A/A | A/A | A/B | B/B | B/B |
| cw159 | B | B | D | B | D | A | A | A/B | A/A | B/B | A/A | A/B | A/B | B/B |
| cw160 | B | B | C | C | C | A | A | A/A | A/A | B/B | A/A | B/B | A/A | A/B |
| ca1 | A | A | B | A | A | A | B | - | - | - | - | - | - | - |
| ca2 | A | A | B | A | A | A | B | - | - | - | - | - | - | - |
| ca3 | A | A | B | A | A | A | B | - | - | - | - | - | - | - |
| ca4 | A | A | B | A | A | A | B | - | - | - | - | - | - | - |
| ca5 | A | A | B | A | A | A | B | - | - | - | - | - | - | - |
| ca6 | A | A | B | A | A | A | B | - | - | - | - | - | - | - |
| ca7 | A | A | B | A | A | A | B | - | - | - | - | - | - | - |
| ca8 | A | A | B | A | A | A | B | - | - | - | - | - | - | - |
| ca9 | A | A | B | A | A | A | B | - | - | - | - | - | - | - |
| ca10 | A | A | B | A | A | A | B | - | - | - | - | - | - | - |
| ca11 | A | A | B | A | A | A | B | - | - | - | - | - | - | - |
| ca12 | A | A | B | A | A | A | B | - | - | - | - | - | - | - |
| ca13 | A | A | B | A | A | A | B | - | - | - | - | - | - | - |
| ca14 | A | A | B | A | A | A | B | - | - | - | - | - | - | - |
| ca15 | A | A | B | A | A | A | B | - | - | - | - | - | - | - |
| ca16 | A | A | B | A | A | A | B | - | - | - | - | - | - | - |
| ca17 | A | A | B | A | A | A | B | - | - | - | - | - | - | - |
| ca18 | A | A | B | A | A | A | B | - | - | - | - | - | - | - |
| ca19 | A | A | B | A | A | A | B | - | - | - | - | - | - | - |
| ca20 | A | A | B | A | A | A | B | - | - | - | - | - | - | - |
| ca21 | A | A | B | A | A | A | B | - | - | - | - | - | - | - |
| ca22 | A | A | B | A | A | A | B | - | - | - | - | - | - | - |
| ca23 | A | A | B | A | A | A | B | - | - | - | - | - | - | - |
| ca24 | A | A | B | A | A | A | B | - | - | - | - | - | - | - |
| ca25 | A | A | C | A | A | A | B | - | - | - | - | - | - | - |

**Table S10 Analysis of molecular variance result.**

| **Locus** | **N** | **Na** | **Ne** | **I** | **Ho** | **He** | **F** |
| --- | --- | --- | --- | --- | --- | --- | --- |
| cwKASP002 | 159 | 2.000 | 1.393 | 0.456 | 0.252 | 0.282 | 0.108 |
| cwKASP003 | 160 | 2.000 | 1.257 | 0.358 | 0.206 | 0.205 | -0.008 |
| cwKASP004 | 160 | 2.000 | 1.969 | 0.685 | 0.450 | 0.492 | 0.086 |
| cwKASP005 | 159 | 2.000 | 1.409 | 0.465 | 0.277 | 0.290 | 0.046 |
| cwKASP007 | 160 | 2.000 | 1.616 | 0.569 | 0.438 | 0.381 | -0.148 |
| cwKASP009 | 160 | 2.000 | 1.768 | 0.626 | 0.400 | 0.434 | 0.079 |
| cwKASP010 | 160 | 2.000 | 1.560 | 0.545 | 0.381 | 0.359 | -0.062 |
| Average | 159.714 | 2 | 1.567 | 0.529 | 0.343 | 0.349 | 0.014 |

Abbreviations: N: sample size, Na: Number of alleles, Ne: effective alleles, I: information index, Ho: Observed heterozygosity, He: Expected heterozygosity, F: Fixation index.
